# Allele-specific depletion of *GNAQ*^Q209L^ via siRNA or an rAAV2-shRNA vector induces selective toxicity in *GNAQ*^Q209L^ uveal melanoma cells

**DOI:** 10.1016/j.omton.2025.201020

**Published:** 2025-07-17

**Authors:** Trace F. McCall, Emma J. Sawyer, Joshua Darnell, Matthew L. Hirsch, Jacquelyn J. Bower

**Affiliations:** 1 Department of Ophthalmology, University of North Carolina at Chapel Hill, Chapel Hill, NC, USA; 2 Carolina Eye Research Institute, University of North Carolina at Chapel Hill, Chapel Hill, NC, USA; 3 Lineberger Comprehensive Cancer Center, University of North Carolina at Chapel Hill, Chapel Hill, NC, USA; 4 Gene Therapy Center, University of North Carolina at Chapel Hill, Chapel Hill, NC, USA

**Keywords:** MT: Regular Issue, uveal melanoma, adeno-associated virus, AAV, cancer gene therapy, GNAQ, G-alpha-q

## Abstract

Approximately 80%–90% of uveal melanomas (UVM) harbor a single base pair substitution in one of two Gα protein subunits (*GNAQ*^Q209L/P^/*GNA11*^Q209L^), resulting in constitutive activation and tumor initiation/progression. Herein, a small interfering RNA (siRNA) that specifically targets *GNAQ*^Q209L^ transcripts induced significant cell death in *GNAQ*^Q209L^ UVM cells, whereas little to no effects were observed on *GNAQ*^wt^ cells or *GNAQ*^wt^ transcripts. The most effective siRNA sequence was subsequently encoded into a short hairpin RNA (shRNA) cassette (shGNAQ^Q209L^), expressed in a recombinant adeno-associated virus (rAAV), and the AAV2 capsid was selected for viral production upon completion of a serotype survey in UVM cells. Transduction with rAAV2-shGNAQ^Q209L^ induced significant cell death in *GNAQ*^Q209L^ cells but not in a *GNAQ*^wt^ UVM line. Unexpectedly, cell death in the *GNAQ*^Q209L^ UVM cells was also observed upon transduction with the non-targeting control rAAV2 (although to a lesser degree than rAAV2-shGNAQ^Q209L^), suggesting that an element of the AAV vector itself exhibits toxicity in *GNAQ*^Q209L^ UVM cells. This work is among the first describing a genetic-based rAAV approach to specifically target an oncogenic mutant driver allele using single base pair allelic discrimination, collectively demonstrating that both siRNA and rAAV methods of *GNAQ*^Q209L^ depletion result in significant UVM cell death.

## Graphical abstract

**Figure:**
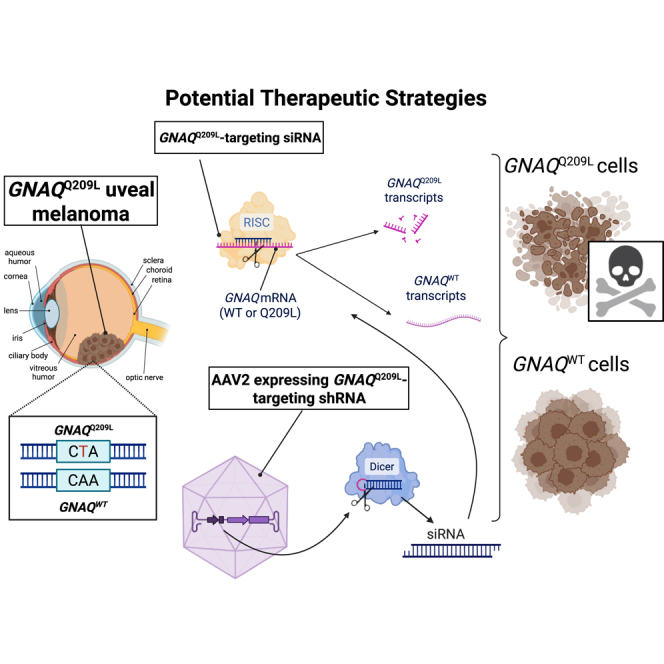

## Teaser

Approximately 80%–90% of uveal melanoma (UVM) patients exhibit a single base pair oncogenic driver mutation in one of two Gα protein subunits (GNAQ/GNA11). siRNA and rAAV approaches that preferentially target a mutant version of GNAQ^Q209L^ were investigated, and both delivery methods induced significant death specifically in GNAQ^Q209L^ UVM cell lines.

## Introduction

Although uveal melanoma (UVM) is considered relatively rare, it is the most common ocular cancer occurring in adults with over 7,000 new diagnoses per year worldwide.^1^^,^^2^ UVM arises from melanocytes present in the uveal tract, a pigmented compartment in the eye composed of the iris, ciliary body, and choroid.^1^ The most common site for primary tumor formation is the choroid (∼90%), and tumors often remain asymptomatic and undetected until they become large enough to cause blurred vision and/or pain.^3^^,^^4^ Local control of primary UVM tumors through radiotherapy and surgical enucleation are often initially successful.^5^^,^^6^^,^^7^ However, approximately 50% of all UVM patients will ultimately be diagnosed with metastatic disease, which exhibits a bleak median survival rate of 4–15 months following the detection of metastatic lesions.^8^^,^^9^^,^^10^^,^^11^^,^^12^

In most other tumors, the number and type of driver mutations are heterogeneous in nature and occur across the mutational spectrum.^13^ However, UVMs are genetically unique in that 80%–90% are initiated by a single base pair substitution on a single allele of one of two Gα protein subunit genes, *GNAQ* or *GNA11* (Gα_q/11_ proteins), in a mutually exclusive pattern.^13^^,^^14^^,^^15^^,^^16^ These Gα_q/11_ mutations occur in one of two highly homologous α subunits of a heterotrimeric G-protein complex that is recruited to the inner cell membrane domain of a G-protein-coupled receptor (GPCR) upon binding to an extracellular ligand.^17^ In normal differentiated melanocytes, this interaction causes the GPCR to transduce the signal across the cell membrane, activating the G protein complex by initiating Gα subunit binding to GTP and the remaining Gβ and Gγ subunits, and inducing cell proliferation and survival signaling.^14^^,^^17^^,^^18^^,^^19^ The Gα subunit then hydrolyzes GTP to GDP, which deactivates the G protein complex and turns off the downstream signaling cascades.^15^^,^^20^^,^^21^

Interestingly, the vast majority of the oncogenic driver mutations occurring in UVMs are located at the *GNAQ/11*^Q209^ amino acid position.^14^^,^^15^ This residue directly associates with GTP, rendering Gα_q/11_ incapable of hydrolyzing GTP that results in a GTPase-defective Gα_q/11_ subunit and constitutive activation of downstream signaling in the absence of GPCR-ligand interactions.^15^^,^^20^^,^^21^ Constitutive activation of Gα_q/11_ further results in sustained nuclear translocation of the yes-associated protein (YAP) and leads to the transcriptional activation of several genes including *CTGF* and *CYR61*, which are associated with anti-apoptotic and angiogenic signaling.^22^^,^^23^^,^^24^^,^^25^^,^^26^^,^^27^^,^^28^^,^^29^

To date, there have been multiple attempts to develop therapeutic interventions for metastatic UVM patients, some of which have focused on directly targeting the mutant Gα_q/11_ pathway.^8^ The small molecule inhibitors FR-900359 (FR) and YM-254890 (YM) have been shown to effectively inhibit wild-type and mutant Gα_q_/Gα_11_/Gα_14_ proteins and downstream signaling, resulting in either UVM cell death *in vitro* or slowed tumor growth *in vivo.*^16^^,^^30^^,^^31^^,^^32^^,^^33^^,^^34^ Unfortunately, FR and YM cannot discriminate between the wild-type and mutant versions of Gα_q_/Gα_11,_ which is prohibitively toxic for systemic delivery.^35^^,^^36^^,^^37^ An alternative approach resulted in a recently approved immunotherapy to treat metastatic UVM (tebentafusp), which extended overall survival in humans for a median of 5 months over current treatments; however, less than half of all metastatic patients are genetically eligible to receive it.^38^^,^^39^^,^^40^ Thus, additional treatment strategies are needed for the majority of metastatic UVM patients.

Because UVM has proven refractory to multiple small molecule inhibitors, chemotherapy, and immune checkpoint blockade strategies, a novel therapeutic approach to specifically target the mutant form of Gα_q_ was investigated.^41^ It was hypothesized that the unique genetic properties of UVM tumor cells could be exploited by generating a small interfering RNA (siRNA) molecule that specifically targeted *GNAQ*^Q209L^ transcripts for degradation while allowing expression of *GNAQ*^wt^ transcripts to minimize potential off-target effects and allow for systemic treatment applications. The work described herein identifies an siRNA sequence that successfully discriminates between *GNAQ*^Q209L^ and *GNAQ*^wt^ transcripts, resulting in *GNAQ*^Q209L^-allele-specific depletion and cell death in two *GNAQ*^Q209L^ human primary tumor-derived UVM cell lines. Neither *GNAQ*^wt^ transcript depletion nor toxicity was observed in a *GNAQ*^wt^ UVM cell line. Toward the development of therapeutic applications, a recombinant adeno-associated virus (rAAV) was subsequently generated with a short hairpin RNA (shRNA) cassette expressing the siRNA sequence targeting *GNAQ*^Q209L^ transcripts, and an AAV serotype survey was performed to identify a capsid suitable for use in UVM cells (rAAV2-shGNAQ^Q209L^). Finally, transduction of *GNAQ*^Q209L^ UVM cells, but not *GNAQ*^wt^ UVM cells, with the AAV2-shGNAQ^Q209L^ vector resulted in significant toxicity. The collective data demonstrate the feasibility of targeting UVM tumors using allele-specific depletion of an oncogenic driver mutant via siRNA- and rAAV-based gene therapy approaches.

## Results

### Design of the *GNAQ*^Q209L^-specific siRNA panel

**Figure 1:**
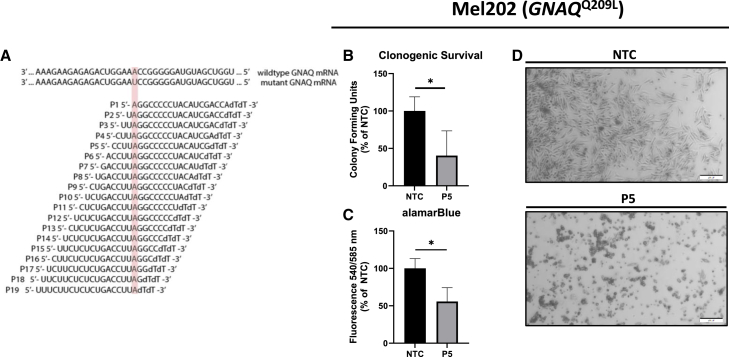
Two siRNA sequences reduced clonogenic survival in a *GNAQ*^Q209L^ UVM cell line (A) *GNAQ*^wt^ and *GNAQ*^Q209L^ transcript sequences are depicted and aligned to 19 different anti-sense *GNAQ*^Q209L^-targeting siRNA sequences. The mutant-targeted/wild-type mismatched nucleotide is highlighted in pink. P1 refers to the Q209L wild-type mismatched nucleotide in the first position relative to the 5′ end, while P19 refers to the nucleotide in the last position relative to the 5′ end of the antisense strand. All 19 siRNAs were examined for their ability to induce toxicity in Mel202 cells (*GNAQ*^Q209L^) via clonogenic survival and alamarBlue fluorescence. (B) Mel202 clonogenic survival on day 20 after non-targeting control siRNA (NTC, *n* = 9) and P5 *GNAQ*^Q209L^-targeting siRNA (P5) transfection (*n* = 12). (C) alamarBlue metabolic activity on day 6 after NTC (*n* = 12) and P5 siRNA transfection (*n* = 12). Fluorescence was measured at excitation 540 nanometers (nm) and emission 585 nm. (D) Representative brightfield microscopy images of bulk transfections with NTC (top) and P5 (bottom) *GNAQ*^Q209L^-targeting siRNA in Mel202 cells, 72 h post-transfection, are depicted. White scale bars overlayed onto microscopy images represent 200 micrometers (μm). Solid bar graphs represent the mean of each dataset, and error bars represent ± the standard deviation (SD) of the mean. Statistical significance was determined using an unpaired t test. Significance levels are indicated by the following: ∗*p* < 0.05.

Previous work has demonstrated that some sequence-specific siRNAs can discriminate between RNA transcripts that differ by a single nucleotide.^42^^,^^43^^,^^44^^,^^45^ Because the majority of oncogenic driver mutations that result in UVM initiation and/or progression occur as a single-base adenine-to-thymine substitution at codon 209 (Q209L) on one allele of *GNAQ* or *GNA11*, it was hypothesized that this unique property could be exploited by designing an siRNA molecule to selectively target *GNAQ*^Q209L^ transcripts while maintaining expression of *GNAQ*^wt^ transcripts. Depletion of *GNAQ*^Q209L^ would be expected to downregulate signaling cascades required for UVM proliferation and survival and ultimately culminate in UVM-specific cell death.^46^ To test this hypothesis, a panel of 19 siRNA sequences were designed to target *GNAQ*^Q209L^ in which the antisense nucleotide of the Q209L mutant was placed in the first position relative to the 5′ end of the siRNA and sequentially shifted toward the 3′ end in each successive molecule, generating a total of 19 different Q209L-targeted sequences (Figure 1A). This creates a mismatch between the mutant-targeting siRNA sequence and wild-type transcript at each position (P) in the siRNA molecule, which was hypothesized to prevent *GNAQ*^wt^ depletion by impeding Dicer-mediated cleavage of the wild-type transcripts as previously demonstrated by Schwarz et al. (Figure 1A).^42^ These siRNA sequences were subsequently examined for the potential to induce cell death in Mel202 cells, a cell line isolated from a primary human uveal melanoma tumor and that harbors a single *GNAQ*^Q209L^ allele.^47^^,^^48^

### Two siRNA sequences reduced clonogenic survival in a UVM cell line harboring *GNAQ*^Q209L^

To examine the impact of *GNAQ*^Q209L^-targeting siRNA on the viability of Mel202 *GNAQ*^Q209L^ UVM cells, transient transfections were performed with each *GNAQ*^Q209L^-targeting siRNA or a non-targeting control (NTC) siRNA labeled with a 6-FAM fluorophore. Twenty-four hours post-transfection, flow cytometry enrichment of the 6-FAM-positive population was performed. alamarBlue metabolic activity, a fluorescence-based indirect measure of cell viability, and clonogenic survival were measured 6 and approximately 14 days post-enrichment, respectively. Overall, colony-forming units (CFUs) were highly variable among *GNAQ*^Q209L^-targeting siRNA-transfected cells compared to NTCs (Figure S1A). Notably, transfection with *GNAQ*^Q209L^-targeting siRNAs containing a mismatch to the wild-type allele at position 2 (P2) and position 5 (P5) significantly decreased clonogenic survival by ∼51% (±19.1%; *p* < .001) and ∼60% (±33.1%; *p* < .001) of the NTC, respectively (Figures 1B and S1A). Consistently, changes in alamarBlue fluorescence were measured 6 days post-enrichment and demonstrated that transfection with either the P2 or P5 *GNAQ*^Q209L^-targeting siRNA sequences reduced metabolic activity (Figure S1B) by ∼18% (±22.4%; *p* = 0.027) and ∼45% (±18.5%; *p* < .001) of the NTC, respectively (Figures 1C and S1B). Representative brightfield microscopy images of Mel202 cells transfected with NTC or the P5 *GNAQ*^Q209L^-targeting siRNA sequence (72-h post-transfection) depict that P5 *GNAQ*^Q209L^-targeting siRNA-treated cells exhibit a detached, aggregated, and non-refractive phenotype indicative of cell death (Figure 1D).^49^ Because the P5 *GNAQ*^Q209L^-targeting siRNA sequence demonstrated the largest reduction in Mel202 cell viability via two independent assays, it was subsequently investigated for its specificity toward the *GNAQ*^Q209L^ transcript.

### P5 *GNAQ*^Q209L^-targeting siRNA preferentially reduces *GNAQ*^Q209L^ transcripts and YAP transcriptional activity

**Figure 2:**
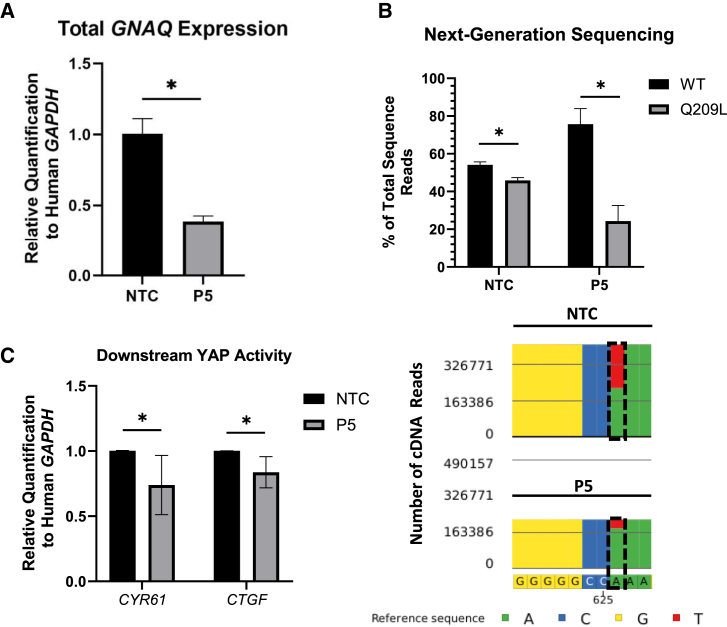
P5 *GNAQ*^Q209L^-targeting siRNA preferentially reduces *GNAQ*^Q209L^ transcripts and YAP transcriptional activity (A) Total *GNAQ* expression measured via reverse-transcription (RT) quantitative PCR between NTC and P5 siRNA-transfected samples, 24 h post-transfection. Data consist of three experimental replicates and three technical replicates each and are normalized to the housekeeping gene human *GAPDH* for each sample and to the NTC data. (B) The results of next-generation Amplicon-EZ sequencing (NGS) of a 263-base pair *GNAQ* cDNA amplicon from NTC and P5 *GNAQ*^Q209L^-targeting siRNA-transfected Mel202 cells are depicted as the percentage of total sequences. Each replicate is a single independent experiment, with a total of five independent experiments performed. Total sequence number varied among samples, with an average of 3.9×10^5^ (±1.0×10^5^) total sequences analyzed per sample. Figure 2B also depicts a representative image of the Partek Flow software analysis, portraying the abundance of recovered *GNAQ* cDNA sequences from NTC and P5 *GNAQ*^Q209L^-targeting siRNA-transfected Mel202 cells. A black box is placed around the mutant nucleotide at position 626 on the *GNAQ* cDNA sequence. Blue = cytosine; yellow = guanine; red = thymine; green = adenine. (C) The functional impacts of the constitutively active Gα_q_ protein in UVM were measured via the relative abundance of *CYR61* and *CTGF* transcripts, which are both transcriptionally activated via the YAP protein, for both NTC- and P5 *GNAQ*^Q209L^-targeting transfected Mel202 cells. Gene expression was normalized to human *GAPDH*. The data shown consist of three independent experiments with three technical replicates each. Error bars represent ± the SD of the mean. Statistical significance was determined using an unpaired t test. Significance levels are indicated by the following: ∗*p* < 0.05.

Due to the reduction in cell viability observed post-transfection with the P5 *GNAQ*^Q209L^-targeting siRNA, *GNAQ* transcript levels were also measured to investigate the siRNA’s specificity for mutant transcripts.^42^ To quantify total *GNAQ* transcripts, RNA was isolated from the 6-FAM-enriched population samples 24 h post-transfection and subjected to RT-qPCR. Residual DNA was not detected in samples that lacked reverse transcriptase (Data Not Shown, DNS). Total *GNAQ*, relative to host *GAPDH*, was depleted by ∼60% (±4%; *p* < .001; Figure 2A) in the P5 *GNAQ*^Q209L^-targeting siRNA-treated cells compared to NTC samples. To characterize individual *GNAQ* transcripts, a 263-base pair region of *GNAQ* cDNA containing codon 209 was amplified by PCR, and next-generation Amplicon-EZ sequencing was performed by Azenta/Genewiz (South Plainfield, NJ, USA).^50^ Allele-specific quantification was completed based on the presence of an adenine (*GNAQ*^wt^) or a thymine (*GNAQ*^Q209L^) nucleotide at base pair position 626 on codon 209 and expressed as a percentage of the total number of sequencing reads. The next-generation sequencing (NGS) results demonstrated that the proportion of *GNAQ*^wt^ to *GNAQ*^Q209L^ transcripts among the NTC samples was approximately 1:1, with 54% containing the wild-type sequence and 46% containing the Q209L sequence (Figure 2B). For Mel202 cells transfected with the P5 *GNAQ*^Q209L^-targeting siRNA, the proportion of *GNAQ*^wt^ to *GNAQ*^Q209L^ significantly increased by a ratio of 3:1, with 76% containing the wild-type sequence and 24% containing the *GNAQ*^Q209L^ mutant sequence (*p* < .001; Figure 2A), suggesting that the P5 *GNAQ*^Q209L^-targeting siRNA preferentially depleted *GNAQ*^Q209L^ transcripts. Allele-specific depletion measurements obtained via Amplicon-EZ NGS were also corroborated by BioRad’s commercially available and wet-lab-validated digital-droplet-mutation-specific PCR assay (Figure S2).

Previous reports have shown that a reduction in both wild-type and mutant *GNAQ* using pooled siRNAs or Gα protein small-molecule inhibitors significantly decrease downstream YAP activity, which then inhibits transactivation of its target genes *CYR61* and *CTGF* in *GNAQ* mutant UVM cell lines.^22^^,^^36^^,^^46^ To characterize the functional impact of P5 *GNAQ*^Q209L^-targeting siRNA depletion, cDNA from samples with confirmed *GNAQ*^Q209L^-specific depletion were subjected to RT-qPCR using primer/probe sets that amplify/detect *CYR61* and *CTGF* cDNA. The P5 *GNAQ*^Q209L^-targeting siRNA significantly reduced both *CYR61* and *CTGF* cDNA abundance, relative to host *GAPDH*, by 26% (±23%; *p* = 0.005; Figure 2C) and 16.3% (±12%; *p* = 0.002; Figure 2C), respectively, 24 hours post-transfection.

### P5 *GNAQ*^Q209L^-targeting siRNA does not affect viability or total *GNAQ* transcript abundance in *GNAQ*^wt^ UVM

**Figure 3:**
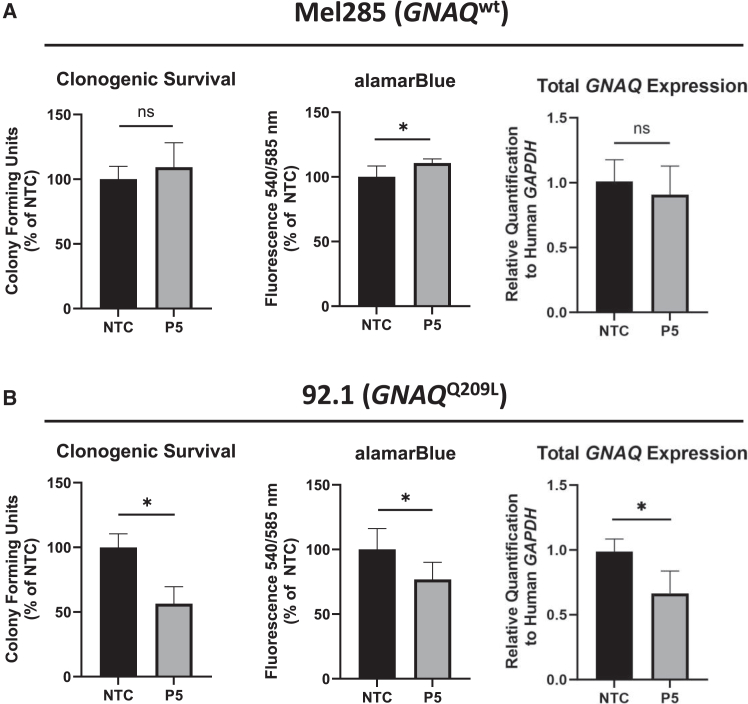
P5 *GNAQ*^Q209L^-targeting siRNA does not affect viability or *GNAQ*^wt^ transcript abundance in *GNAQ*^wt^ UVM (A) P5 *GNAQ*^Q209L^-targeting siRNA transfections of Mel285 (*GNAQ*^wt^) UVM cells are shown for clonogenic survival (*n* = 17), alamarBlue metabolic activity (*n* = 18), and RT-qPCR of total *GNAQ* expression (*n* = 9). (B) P5 *GNAQ*^Q209L^-targeting siRNA transfection of 92.1 (*GNAQ*^Q209L^) UVM cells are shown for clonogenic survival (*n* = 22), alamarBlue metabolic activity (*n* = 20), and RT-qPCR of total *GNAQ* expression (*n* = 9). Clonogenic survival and alamarBlue metabolic activity data consist of at least three independent experiments with at least four technical replicates each. RT-qPCR data consist of three experimental replicates and three technical replicates. All data are normalized to the mean of the NTC. Solid bars represent the mean of each dataset, and error bars represent ± the SD of the mean. Statistical significance was determined using an unpaired t test. Significance levels are indicated by the following: ns, not significant; ∗*p* < 0.05.

To ensure that the effects of the P5 *GNAQ*^Q209L^-targeting siRNA observed in the Mel202 cell line were due to its *GNAQ*^Q209L^ mutation status, similar clonogenic survival and alamarBlue assays were performed on two additional previously characterized human UVM cell lines, Mel285 (*GNAQ*^wt^) and 92.1 (*GNAQ*^Q209L^).^48^^,^^51^^,^^52^ Flow cytometry enrichment of siRNA-transfected cell populations was completed for both Mel285 and 92.1 in the same manner as described for Mel202 cells. The P5 *GNAQ*^Q209L^-targeting siRNA failed to reduce viability in Mel285 cells in either assay (Figure 3A). Although a slight increase in alamarBlue fluorescence (110.7% ± 3.2%; *p* = 0.001; Figure 3A) was observed in Mel285 cells transfected with the P5 *GNAQ*^Q209L^-targeting siRNA, there was no difference between these samples via clonogenic survival (*p* = 0.132; Figure 3A). In addition, P5 *GNAQ*^Q209L^-targeting siRNA did not alter total *GNAQ* transcripts in Mel285 cells (*p* = 0.347, Figure 3A). Conversely, transfection with the P5 *GNAQ*^Q209L^-targeting siRNA in the 92.1 *GNAQ*^Q209L^ cell line significantly reduced clonogenic survival and alamarBlue fluorescence by 43.0% (±13.7%; *p* < .001; Figure 3B) and 23.2% (±13.4%; *p* < .001; Figure 3B), respectively. Partial depletion of total *GNAQ* transcripts in 92.1 cells was also confirmed through RT-qPCR and was significantly reduced by ∼33% (±17%, *p* < .001, Figure 3B). Collectively, these data demonstrate that the reduction in cell viability after treatment with the P5 *GNAQ*^Q209L^-targeting siRNA occurred in multiple *GNAQ*^Q209L^ cell lines and failed to affect *GNAQ* transcripts in *GNAQ*^wt^ cells.

### Adeno-associated virus serotype 2 efficiently transduces UVM cell lines

**Figure 4:**
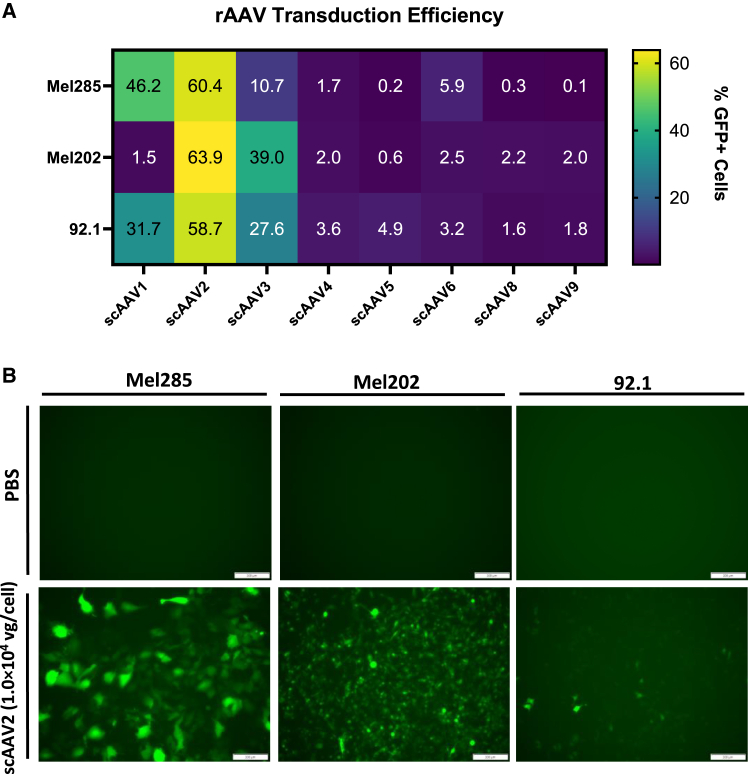
Adeno-associated virus serotype 2 (AAV2) efficiently transduces UVM cell lines (A) Mel285, Mel202, and 92.1 human UVM cell lines were transduced with self-complementary adeno-associated virus (scAAV) preparations of capsid serotypes 1, 2, 3, 4, 5, 6, 8, and 9 at 1.0×10^4^ viral genomes per cell (vg/cell) containing a green fluorescent protein reporter transgene (GFP) under control of the ubiquitous cytomegalovirus promoter (CMV). Transduction efficiency is represented by the percentage of GFP^+^ cells and was quantified via flow cytometry 72 h post-transduction. The mean of at least four replicates is displayed in a heatmap. Cell lines are displayed on the *y* axis, and AAV serotypes are displayed on the *x* axis. The color key corresponding to the percentage of cells that were GFP^+^ is displayed on the right. (B) Representative GFP fluorescence microscopy images are displayed for scAAV2-transduced Mel285, Mel202, and 92.1 cell lines. Images shown were taken 72 h post-transduction of 1.0 × 10^4^ vg/cell scAAV2-CMV-GFP (bottom) and vehicle control, phosphate-buffered saline (PBS; top). White scale bars overlayed onto microscopy images represent 200 micrometers (μm).

Due to the short half-life of siRNA within cells and for a more efficient delivery method to UVM cells, an adeno-associated virus (AAV) strategy was pursued with the intent to constitutively express the P5 *GNAQ*^Q209L^-targeting siRNA sequence as an shRNA cassette.^53^^,^^54^^,^^55^^,^^56^^,^^57^ Although a multitude of AAV capsid serotypes have been isolated from several species that exhibit selective tropism for tissue-specific transduction,^58^ a comprehensive analysis regarding AAV transduction efficiency in UVM cell lines has not been reported to date. Thus, the transduction efficiency of eight natural AAV capsid serotypes (AAV1-6, 8, and 9) was investigated. AAV capsids were packaged with a self-complementary (sc) vector genome encoding the green fluorescent protein (GFP) reporter transcribed from the ubiquitous cytomegalovirus (CMV) promotor (scAAV-CMV-GFP).^59^ All three UVM cell lines utilized in this study (Mel285, Mel202, and 92.1) were transduced with each of the eight serotypes at 1.0 × 10^4^ viral genomes per cell (vg/cell), and the percentage of GFP^+^ cells was measured via flow cytometry 3 days post-transduction. Of all serotypes examined, serotype 2 (scAAV2-CMV-GFP) transduced all tested UVM cell lines with the highest efficiency (Figures 4A and S3). On average, 60% (±14%) of Mel285 cells, 64% (±1.3%) of Mel202 cells, and 59% (±4.0%) of 92.1 cells were GFP^+^ (Figures 4A and S3). Furthermore, rAAV1 and rAAV3 transduced UVM cell lines with varying efficiencies, while little to no transduction was observed using rAAV4-6, 8, and 9 capsids (Figures 4A and S3). Representative GFP fluorescence microscopy images of rAAV2-transduced and PBS control Mel285, Mel202, and 92.1 cells are shown in Figure 4B.

### rAAV2-shGNAQ^Q209L^ vector design

**Figure 5:**
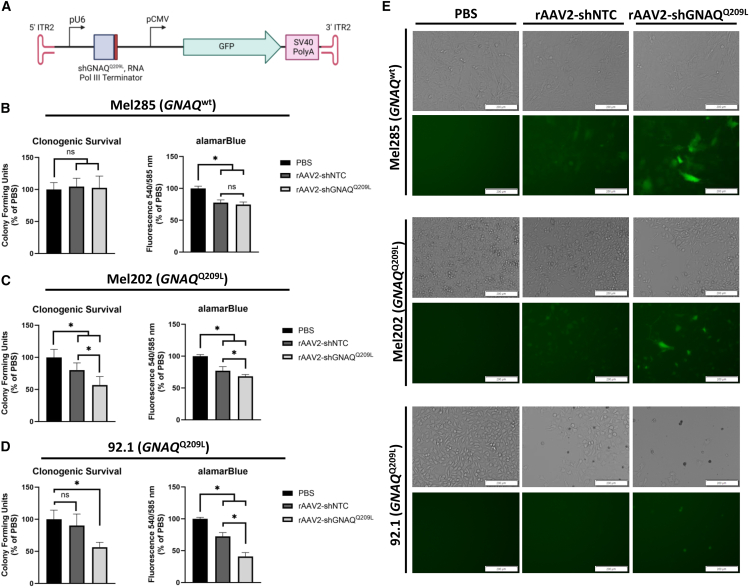
rAAV2-shGNAQ^Q209L^ transduction results in *GNAQ*^Q209L^ UVM cell death, similar to that observed with P5 *GNAQ*^Q209L^-targeting siRNA (A) The *cis*-regulatory elements and genetic cassettes of the single-stranded (ss) AAV vector are depicted (ITR, inverted terminal repeat; pU6, U6 RNA polymerase III promoter; pCMV, cytomegalovirus promoter; GFP, green fluorescent protein; SV40, simian virus 40). Black arrows indicate promoter sequences, direction, and location, and the large green arrow indicates the GFP open reading frame. Solid rectangles represent other coding and non-coding elements. Clonogenic survival and alamarBlue metabolic activity are shown for PBS (black bars, vehicle control) and 1.0×10^4^ vg/cell of rAAV2-shNTC (dark gray bars) or rAAV2-shGNAQ^Q209L^ (light gray bars) for (B) Mel285 (*GNAQ*^wt^), (C) Mel202 (*GNAQ*^Q209L^), and (D) 92.1 (*GNAQ*^Q209L^) cell lines. Cells were analyzed 7 days post-transduction of vectors for alamarBlue metabolic activity and approximately 14 days post-transduction of vectors for clonogenic survival. Data consists of 2–3 independent experiments with at least four technical replicates each and was normalized to the mean of PBS. (E) Representative brightfield and GFP fluorescence microscopy images for PBS (left), rAAV-shNTC (middle), and rAAV-shGNAQ^Q209L^ (right)-treated Mel285 (top), Mel202 (middle), and 92.1 (bottom) cells are shown 6 days following PBS and vector addition. White scale bars overlayed onto microscopy images represent 200 micrometers (μm). Solid bars represent the mean of each dataset, and error bars represent ± the SD of the mean. Statistical significance was determined using an unpaired t test. Significance levels are indicated by the following: ns, not significant; ∗*p* < 0.05.

Because rAAV2 transduced all tested UVM cell lines with the highest efficiency *in vitro*, the P5 *GNAQ*^Q209L^-targeting siRNA sequence was converted to an shRNA cassette and vectorized for delivery via rAAV2 in a single-stranded (ss) genome context (Figure 5A). Following the flanking 5′ inverted terminal repeat of serotype 2 (ITR2), the P5 *GNAQ*^Q209L^-targeting shRNA cassette was placed under the control of the U6 promotor, and an RNA polymerase III terminator sequence was added to the 3′ end.^60^ Downstream of the shRNA cassette, the GFP open reading frame (ORF) was placed under the control of the CMV promoter and the simian virus 40 (SV40) poly A tail as a separate expression cassette followed by the 3′ flanking ITR. Henceforth, this vector is referred to as rAAV2-shGNAQ^Q209L^ (Figure 5A). A near identical control vector was also generated that replaced the P5 *GNAQ*^Q209L^-targeting siRNA sequence with the NTC control sequence (rAAV2-shNTC). The NTC sequence was confirmed to lack sequence similarity to any known human or mouse RNA sequences, with at least seven mismatches between the NTC sequence and other known messenger RNAs via a National Center for Biotechnology Information (NCBI) BLAST search.^61^ Both rAAV2-shGNAQ^Q209L^ and rAAV2-shNTC were obtained from VectorBuilder (Chicago, IL, USA), and the viral preparations were characterized via alkaline gel electrophoresis to confirm packaged vector size and qPCR to determine viral genome titer (Figure S4). SYBR Gold staining of packaged vector genomes revealed that the majority of packaged species were single-stranded, as expected; however, some suspected self-complementary AAV (scAAV) genomes were noted, which is unsurprising, given the approximately 2.2 kB size of the transgenic genome (Figure S4).^59^

### rAAV2-shGNAQ^Q209L^ transduction results in *GNAQ*^Q209L^ UVM cell death, similar to the P5 *GNAQ*^Q209L^-targeting siRNA

To assess the effects of the vectorized P5 *GNAQ*^Q209L^-targeting sequence, Mel285, Mel202, and 92.1 cells were again subjected to clonogenic survival and alamarBlue metabolic activity assays. For clonogenic survival, 1.0 × 10^3^ single UVM cells were plated in cell culture dishes, and the following day, equal volumes of vehicle control (PBS) or 1.0 × 10^4^ vg/cell of rAAV2-shNTC or rAAV2-shGNAQ^Q209L^ was added to each culture (Figures 5B–5D). Approximately 14 days post-transduction, clonogenic survival of *GNAQ*^wt^ Mel285 was not significantly different after treatment with rAAV2-shNTC or rAAV2-shGNAQ^Q209L^ (*p* = 0.725; Figure 5B). However, rAAV2-shGNAQ^Q209L^ transduction significantly reduced colony formation of Mel202 and 92.1 cells by ∼43% (±12.6%; *p* < .001) and ∼44% (±7.3%; *p* < .001), respectively, compared to PBS (Figures 5C and 5D). Surprisingly, rAAV2-shNTC transduction also reduced clonogenic survival, although to a lesser extent, in Mel202 and 92.1 cells, by ∼20% (±10.5%; *p* = 0.002) and ∼10% (±17%; *p* = 0.193), respectively (Figures 5C and 5D). To determine the time point at which optimal cell death was observed post-rAAV2-shGNAQ^Q209L^ transduction, a time course experiment comparing the impact of both vectors on alamarBlue fluorescence was performed, and data are shown in Figure S5. All three UVM cell lines were subsequently assessed for alamarBlue metabolic activity after addition of PBS or 1.0 × 10^4^ vg/cell of either shRNA vector 7 days post-transduction (Figures 5B–5D). The rAAV2-shGNAQ^Q209L^ vector reduced fluorescence in Mel202 and 92.1 UVM cell cultures by an average of ∼32% (±2.8%; *p* > 0.001) and ∼61% (±6.2%; *p* < 0.001), respectively (Figures 5C and 5D). Similar to Mel202 and 92.1 colony formation, the rAAV2-shNTC vector also reduced fluorescence by ∼23% in Mel202 cells (±6.3%; *p* > 0.001) and ∼28% in 92.1 cells (±6.1%; *p* > 0.001) (Figures 5C and 5D). Both rAAV2-shNTC and rAAV2-shGNAQ^Q209L^ slightly reduced fluorescence in Mel285 cells by ∼22% (±4.1%; *p* < 0.001) and ∼25% (±3.9%; *p* < 0.001), respectively, but there was no significant difference between the vectors (Figure 5B).

Representative images of both brightfield and GFP fluorescence microscopy indicate that Mel285 cells appear normal and exhibit similar morphology across treatments; however, vector-treated Mel202 and 92.1 cells show a reduction in the number of adherent cells post-transduction of both rAAV2-shNTC and rAAV2-GNAQ^Q209L^, with rAAV2-GNAQ^Q209L^ demonstrating the largest reduction in viability (Figure 5E). Although approximately 60% of UVM cells are transduced *in vitro*, as demonstrated in Figure 4, the data herein suggest a majority of rAAV-shGNAQ^Q209L^-transduced cells died prior to analysis (Figures 5 and S5). The dark, small, and circular objects visible in the brightfield images exhibit autofluorescence in both Mel202 and 92.1 cells, possibly indicating the release of melanin or melanosomes following uveal melanoma cell death (Figure 5E, middle and bottom).^62^^,^^63^ These morphological changes were not observed in the *GNAQ*^wt^ Mel285 cells (Figure 5E, top panels). Taken together, these data suggest that the rAAV2-shGNAQ^Q209L^ vector induced cell death in *GNAQ*^Q209L^ UVM cells, and, to a lesser extent, the rAAV2-shNTC vector also induced *GNAQ*^Q209L^ UVM cell death.

## Discussion

For UVM patients diagnosed with liver metastases, there are very few treatment options, and the outcome is an almost 100% fatality rate within 6–12 months.^3^^,^^4^^,^^64^^,^^65^^,^^66^ Previous efforts to target the oncogenic driver mutations responsible for UVM initiation and survival have proven challenging, often resulting in systemic toxicity *in vivo* due to an inability to distinguish between the mutant and wild-type forms,^32^^,^^33^^,^^35^^,^^36^^,^^37^ and targeting of the Gα_q_ pathway at downstream signaling nodes has thus far proven ineffective.^67^ Here, evidence is presented demonstrating that, first, an siRNA molecule designed to target the *GNAQ*^Q209L^ driver mutation can selectively deplete *GNAQ*^Q209L^ transcripts while maintaining at least a 3-fold higher ratio of *GNAQ*^wt^ transcripts, ultimately resulting in significant cell death in multiple *GNAQ*^Q209L^ UVM cell lines established from different primary human tumors (Figures 1, 2, and 3). In a *GNAQ*^wt^ cell line, transfection with the P5 *GNAQ*^Q209L^-targeting siRNA did not lead to a significant reduction in total *GNAQ* transcripts nor did it result in cell death, illustrating the selectivity of the P5 *GNAQ*^Q209L^-targeting siRNA. A second approach employing an shRNA cassette based on the P5 *GNAQ*^Q209L^-targeting sequence delivered via an AAV vector (rAAV2-shGNAQ^Q209L^) also induced cell death in multiple *GNAQ*^Q209L^ cell lines (Figures 4 and 5). Taken together, these data suggest that allele-specific depletion of *GNAQ*^Q209L^ transcripts via siRNA or an shRNA cassette coupled with an AAV delivery vector is a feasible and promising gene therapy approach for the treatment of UVM.

One of the most significant aspects of the work presented herein is the demonstration that *GNAQ*^Q209L^ UVM cell survival is dependent on *GNAQ*^Q209L^ transcript expression, which can be exploited to induce cell death. Importantly, this approach directly targets the mutant form of a Gα protein, which has historically been considered an undruggable target. Nineteen different siRNA sequences were initially screened to identify sequences that could reduce *GNAQ*^Q209L^ UVM cell survival (Figure 1), and, ultimately, the P2 and P5 *GNAQ*^Q209L^-targeting siRNAs were the only sequences that reduced cell viability (Figure S1). The siRNA’s positional- and sequence-specific effects described herein are consistent with the findings of Schwarz et al., likely because the position of the mismatched base pair occurs at the RNA-induced silencing complex (RISC) cleavage site, thus allowing the wild-type mRNA to escape degradation.^42^^,^^68^^,^^69^^,^^70^^,^^71^^,^^72^ The P5 *GNAQ*^Q209L^-targeting siRNA was chosen for further characterization, as this sequence induced the largest percentage of cell death in Mel202 cells (Figures 1 and S1). Although all siRNAs tested were antisense matches to the *GNAQ*^Q209L^ mRNA, it was unexpectedly observed that approximately half of the *GNAQ*^Q209L^*-*specific siRNAs resulted in significant increases in both clonogenic survival and alamarBlue fluorescence compared to the NTC siRNA (Figure S1). An NCBI BLAST search of all 19 siRNAs against known transcript sequences in the human and mouse genomes suggest they have no other exact sequence matches, but several of the duplexed siRNAs contained at least some sequence complementarity to other mRNAs that may affect off-target transcript regulation of cell metabolism and/or proliferation.^61^^,^^73^^,^^74^^,^^75^^,^^76^^,^^77^ Although this variability is interesting, these mechanisms are not fully understood and are beyond the scope of the current study.

A second important aspect of this work is the demonstration that preferential depletion of a single-nucleotide mutant transcript can be achieved while preserving wild-type transcript expression. Figure 2 demonstrates that a ∼60% reduction in total *GNAQ* transcripts at 24 h post-transfection of P5 *GNAQ*^Q209L^-targeting siRNA results in a 3:1 *GNAQ*^Q209L^-selective bias in transcript depletion and is observed in conjunction with a significant reduction in the YAP-induced transactivation of *CTGF* and *CYR61* genes. It has previously been shown that *GNAQ*^wt^ expression remains necessary for normal cell signaling and function: defects in platelet activation and mouse development/cognition have been observed in *GNAQ* ^−/−^ knockout models, and systemic treatment of mice with Gα_q/11_ small molecule inhibitors results in acute toxicity.^16^^,^^30^^,^^31^^,^^35^^,^^36^^,^^78^^,^^79^ Taken together, the data in Figures 1 and 2 demonstrate that the P5 *GNAQ*^Q209L^-targeting siRNA sequence preferentially discriminated between wild-type and mutant *GNAQ* transcripts while functionally reducing aberrant downstream signaling associated with UVM survival/progression. This suggests that a genetic-based approach that preferentially targets *GNAQ*^Q209L^ transcripts may provide an alternative therapeutic strategy for UVM with fewer off-target side effects.

To confirm that the P5 *GNAQ*^Q209L^-targeting siRNA preferentially targeted *GNAQ*^Q209L^ transcripts, a *GNAQ*^wt^ UVM cell line, Mel285, was assayed to determine whether the P5 *GNAQ*^Q209L^-targeting siRNA could also deplete *GNAQ*^wt^ transcripts. As expected, Mel285 cells exhibited neither a significant reduction in cell survival nor a change in total *GNAQ* transcripts when treated with the P5 *GNAQ*^Q209L^-targeting siRNA (Figure 3A). Although a small increase in alamarBlue fluorescence was observed following P5 *GNAQ*^Q209L^-targeting siRNA transfection in Mel285s, neither an increase in clonogenic survival nor an increase in total *GNAQ* transcripts were observed, suggesting the increased metabolic activity was not the result of upregulation of total *GNAQ* expression (Figure 3B).^80^^,^^81^ To confirm that the decrease in UVM cell viability and significant *GNAQ*^Q209L^ transcript depletion observed in the Mel202 cell line were due to its *GNAQ*^Q209L^ mutation status, identical experiments were performed in an additional *GNAQ*^Q209L^ UVM cell line, 92.1.^82^ Indeed, similar to the Mel202 cell line, transfection with P5 *GNAQ*^Q209L^-targeting siRNA significantly reduced cell survival in 92.1 cells (Figure 3B), and total *GNAQ* transcript abundance was depleted by roughly 33% (Figure 3B). Furthermore, NGS demonstrated that the 1:1 ratio of wild-type to mutant GNAQ was increased to 2:1 post-treatment with the P5 *GNAQ*^Q209L^-targeting siRNA (Figure S2). Taken together, these data suggest that the P5 *GNAQ*^Q209L^-targeting siRNA sequence consistently depletes *GNAQ*^Q209L^ transcripts in multiple *GNAQ*^Q209L^ UVM cell lines, which results in cell death.

Upon demonstrating that an siRNA-based delivery approach using the P5 *GNAQ*^Q209L^-targeting sequence induced cell death in G*NAQ*^Q209L^ UVM cells by preferentially depleting *GNAQ*^Q209L^ transcripts, a second delivery method utilizing the most commonly employed gene therapy vector, rAAV, was also explored to determine whether an increase in the durability of the P5 *GNAQ*^Q209L^-targeting sequence would enhance its toxicity in *GNAQ*^Q209L^ UVM cells.^43^^,^^77^^,^^83^^,^^84^^,^^85^^,^^86^ This hypothesis was tested by vectorizing the P5 *GNAQ*^Q209L^-targeting siRNA sequence into an shRNA cassette for continuous expression and delivered via an AAV2 viral vector. rAAV was chosen as the vector because the US Food and Drug Administration (FDA) has approved multiple AAV-based therapies for clinical use, highlighting their excellent safety profile and capacity for long-term transgene expression.^77^^,^^87^^,^^88^^,^^89^^,^^90^^,^^91^^,^^92^^,^^93^^,^^94^^,^^95^^,^^96^ An AAV serotype transduction survey of the Mel285, Mel202, and 92.1 UVM cell lines demonstrated that AAV serotype 2 transduced all three UVM cell lines with high efficiency *in vitro* (Figure 4). These results were not surprising as previous reports have shown that human melanoma cell lines exhibit upregulated heparan sulfate proteoglycan and human fibroblast growth factor receptor 1, two identified membrane surface proteins that aid in the intracellular uptake of AAV2.^97^^,^^98^^,^^99^ A serotype survey comparing AAV transduction between primary human melanocytes and human cutaneous melanoma cell lines demonstrated that AAV6 transduced primary cutaneous melanocytes with the highest efficiency, but AAV2 exhibited the highest transduction efficiency in melanoma cell lines.^100^ Thus, for future pre-clinical applications, multiple serotypes will need to be to be examined to empirically determine which capsid exhibits the highest transduction efficiencies for UVM cells *in vivo*.

Successful AAV-mediated delivery of shRNA cassettes has previously been shown to selectively induce cancer cell death through transcript depletion in multiple other types of cancer *in vivo.*^56^^,^^101^ However, to our knowledge, this report is the first to describe an rAAV-shRNA-based approach targeting a single base pair driver mutation on one allele in UVM. The rAAV2-shGNAQ^Q209L^ vector was generated to deliver the *GNAQ*^Q209L^-targeting shRNA cassette while simultaneously expressing GFP to monitor transduction (Figure 5A). Although significant reductions in clonogenic survival and alamarBlue fluorescence were observed with rAAV2-shGNAQ^Q209L^ transduction in Mel202 and 92.1 UVM cells, alamarBlue fluorescence was slightly decreased in Mel285 cells (Figures 5B–5D and S5). Unexpectedly, the control vector (rAAV2-shNTC) also exhibited some toxicity in the *GNAQ*^Q209L^ UVM cell lines, although to a significantly lesser extent than rAAV2-shGNAQ^Q209L^. These data suggest that a property of rAAV and/or the transgene cassette induces cell death in *GNAQ*^Q209L^ UVM cells independent of *GNAQ*^Q209L^ transcript depletion. We and others have previously observed this phenomenon with wild-type AAV and rAAV in certain types of cancer cells and stem cells, which has been attributed, at least in part, to the AAV ITR sequence (Figures 5 and S5).^102^^,^^103^^,^^104^^,^^105^^,^^106^^,^^107^^,^^108^^,^^109^^,^^110^ Although not well understood, the mechanism(s) of ITR-induced cell death in a subset of cancer cells is an area of active investigation in the laboratory, and we hope to shed some light on this area in future publications.

Although the Mel285 cell line exhibited decreased alamarBlue fluorescence upon transduction with both the control rAAV2-shNTC and rAAV2-shGNAQ^Q209L^ compared to the PBS control, the difference between the NTC and *GNAQ*^Q209L^-specific virus was not significant (Figures 5B, 5E, and S5). Furthermore, there were no alterations in Mel285 clonogenic survival, Mel285 cell morphology upon transduction with either the control or *GNAQ*^Q209L^-specific virus, or the release of melanin and/or melanosomes observed in these cultures, suggesting that the Mel285 UVM cell line likely experienced slower cell growth and/or metabolism upon rAAV transduction as measured by alamarBlue (Figures 5B, 5E, and S5). In Figure 5E, microscopic analysis also confirms the abundance and similarity of adherent Mel285 cells in PBS and rAAV-transduced cells. In contrast, Mel202 and 92.1 cells exhibit both decreased adherent cell number and increased levels of dark colored and circular debris throughout the culture media across rAAV-transduced wells, similar to melanin-like particles that are often released upon melanocyte cell death.^62^^,^^63^ Although Mel202 and 92.1 cell death was incomplete following rAAV2-shGNAQ^Q209L^ transduction, the amount of cell death observed was proportional to the transduction efficiency measurements for each cell line. This implies that rAAV2-shGNAQ^Q209L^ effectively eliminated transduced *GNAQ*^Q209L^ cells but was unable to transduce all UVM cells *in vitro* (Figures 4, 5C–5E, and S5). This is further exemplified by the lack of GFP fluorescence in rAAV-transduced 92.1 cells noted in the right panels of Figure 5E, suggesting that most, if not all, GFP^+^ 92.1 cells died prior to analysis. Taken together, these data suggest that rAAV2-shGNAQ^Q209L^ results in cell death in *GNAQ*^Q209L^ UVM cells, similar to the effects observed with P5 *GNAQ*^Q209L^-targeting siRNA sequences, providing a path forward for the optimization of an AAV-based therapeutic strategy.^102^^,^^103^^,^^104^

In summary, this report provides proof of concept that the P5 *GNAQ*^Q209L^-targeting siRNA significantly reduced *GNAQ*^Q209L^ transcripts at least 3-fold over *GNAQ*^wt^, resulting in effective discrimination between *GNAQ*^wt^ and *GNAQ*^Q209L^ transcripts and *GNAQ*^Q209L^-specific cell death. Although both rAAV2-shGNAQ^Q209L^ and rAAV2-shNTC reduced alamarBlue fluorescence measurements of metabolic activity and clonogenic survival, rAAV2-shGNAQ^Q209L^ resulted in a significantly higher amount of toxicity in *GNAQ*^Q209L^ UVM cell lines, suggesting that the *GNAQ*^Q209L^-targeting shRNA cassette induces cell death similar to that observed with the P5 *GNAQ*^Q209L^-targeting siRNA. Follow-up studies will examine a similar approach to *GNA11*^Q209L^ mutant UVM and assess the *in vivo* efficacy of rAAV2-shGNAQ^Q209L^ delivery in a previously characterized mouse model of metastatic *GNAQ*^Q209L^ UVM.^111^ The mechanisms of AAV-specific toxicity observed here with shNTC vectors remain elusive and will also be examined in future work.

## Materials and methods

### Cell culture

Mel202 (CVCL_C301) and 92.1 (CVCL_8607) human UVM cell lines were purchased from Sigma-Aldrich Inc. (Saint Louis, MO, USA). The Mel285 (CVCL_C303) human UVM cell line was a kind gift from Dr. Martine Jaeger (Leiden University, Leiden, the Netherlands). *GNAQ/11* mutation status was confirmed via Sanger sequencing (Azenta/Genewiz, South Plainfield, NJ, USA) for all three UVM cell lines (DNS). Each cell line’s STR profile was also confirmed, and periodic mycoplasma testing remained negative throughout the study (DNS). Cell lines were cultured with RPMI Medium 1x (Gibco, Grand Island, NY, USA), 10% fetal bovine serum (Omega Scientific Inc., Tarzana, CA, USA), 2 mM L-glutamine (Gibco, Grand Island, NY, USA), and 1% antibiotic-antimycotic solution (Gibco, Grand Island, NY, USA) at 5% CO_2_ and 37°C. UVM culture media was replaced every 2–3 days and sub-cultured at approximately 60%–80% confluency.

### siRNA transfections

All siRNA sequences targeting *GNAQ*^Q209L^ transcripts were designed as previously described by Schwarz et al.^42^ The NTC siRNA was purchased from MilliporeSigma (Burlington, MA, USA) and does not target any known sequence in the human or mouse genome. Transfections of UVM cells were completed with 800 pmol of each siRNA in an 80% confluent 15-cm cell culture dish using a Lipofectamine RNAiMAX kit (Invitrogen/Thermo Fisher Scientific Inc., Waltham, MA, USA) following the manufacturer’s instructions. siRNA sequences were custom ordered from MilliporeSigma (Burlington, MA, USA), conjugated to a 6-FAM fluorophore, and are listed in Figure 1A. Each siRNA sequence also contained a deoxythymidine dinucleotide (dTdT) overhang to enhance intracellular stability (Figure 1A).

### Flow cytometry cell enrichment

Transfected UVM cell populations were enriched for 6-FAM^+^ cells 24 h following transfection of siRNA using the Becton Dickinson FACSAria II (BD Biosciences, Franklin Lakes, NJ, USA) system operated by the UNC Flow Cytometry Core Facility (UNC-Chapel Hill, Chapel Hill, NC, USA). Propidium iodide was used as a live/dead discrimination dye (Invitrogen/Thermo Fisher Scientific Inc., Waltham, MA, USA); 100,000 6-FAM^+^ live UVM cells were collected for each sample and subsequently divided for clonogenic survival, alamarBlue assays, and reverse-transcription reactions for RT-qPCR and NGS.

### siRNA-based clonogenic survival assay

One thousand single live 6-FAM^+^ cells were seeded onto 10-cm tissue culture dishes in replicates of at least four for each experimental group. Cultures were fed with fresh media every 2–3 days for 18–20 days until colonies containing ≥50 cells became visible. Media was removed from each plate, stained with a 0.05% crystal violet/40% methanol solution, washed with phosphate-buffered saline (PBS) solution, and dried overnight. Plates were imaged using the Amersham ImageQuant 800 system (Cytiva, Marlborough, MA, USA) and analyzed for colony numbers with a hand count on the ImageQuant TL analysis software (Cytiva, Marlborough, MA, USA). The data shown are compiled from at least three independent experiments with at least four technical replicates each.

### siRNA-based alamarBlue assay

Two thousand live 6-FAM^+^ cells were seeded in each well of a 96-well plate in replicates of four. Six days following plating, alamarBlue HS Cell Viability Reagent (Life Technologies Corporation, Eugene, OR, USA) was added to each well and incubated/monitored for up to 24 h at a final concentration of 10% of the medium volume. Cellular metabolism of the alamarBlue dye was verified through visual color changes in the solution, and fluorescence readings were taken 2, 4, and 6 hours post-addition of the dye. Fluorescence (Excitation: 540/20 nm; Emission: 585/20 nm) of each well was quantified on the BioTek Cytation 5 Imaging Reader system (Lineberger Comprehensive Cancer Center, UNC-Chapel Hill, NC, USA; Agilent, Santa Clara, CA, USA). The data shown are compiled from at least three independent experiments with at least four technical replicates each.

### RT-qPCR

To quantify total *GNAQ*, *CTGF*, *CYR61*, and *GAPDH* transcript levels, RT-qPCR was performed in triplicate for each sample. RNA was isolated from flow-cytometry-enriched 6-FAM^+^ cell pellets with a RNeasy kit (Qiagen, Hilden, Germany) and treated with a DNA-*free* DNA removal kit (Invitrogen/Thermo Fisher Scientific Inc., Waltham, MA, USA). First-strand cDNA synthesis was performed using the High-Capacity cDNA Reverse Transcription kit (Applied Biosystems/Thermo Fisher Scientific Inc., Waltham, MA, USA) according to the manufacturer’s instructions. For qPCR analysis, a 2x TaqMan Universal PCR Master Mix (Applied Biosystems/Thermo Fisher Scientific Inc., Waltham, MA, USA) was used with TaqMan 20x gene expression assay primer/probe mixes for each target analyzed in a total reaction volume of 20 μL/well. Reactions were run on the StepOnePlus Real-Time PCR System (Applied Biosystems/Thermo Fisher Scientific Inc., Waltham, MA, USA) under relative quantification with the following thermal cycling parameters: 50°C for 2 minutes and 95°C for 10 minutes followed by 40 cycles of 95°C for 15 seconds and 60°C for 1 minute. TaqMan assay numbers are listed in Table S1. Relative quantification of gene expression to Human *GAPDH* was calculated as previously described.^112^ RNA samples not subjected to reverse transcription were also analyzed through RT-qPCR to ensure the absence of contaminating residual DNA.

### Next-generation sequencing

NGS was used to differentiate the relative number of *GNAQ*^Q209L^ and *GNAQ*^wt^ cDNA copies in each sample. Following cDNA synthesis, the region of interest, a 263-base pair amplicon containing *GNAQ*^209^, was subject to PCR amplification using the Phusion High Fidelity PCR kit (New England Biolabs, Ipswich, MA, USA). Amplified cDNA of NTC and P5 *GNAQ*^Q209L^-targeting siRNA-transfected samples were briefly purified using a QIAquick PCR Purification kit following the manufacturer’s instructions (Qiagen, Hilden, Germany). PCR amplification was confirmed based on the presence of 263-base pair amplicons on a 2% agarose gel. Samples were then shipped to Azenta/Genewiz (Azenta Life Sciences, South Plainfield, NJ, USA) for Amplicon-EZ sequencing. The Partek Flow Software (Partek Incorporated, Chesterfield, MO, USA) was used for analysis of returned sequences. Sequences were aligned with a Burrows-Wheeler Alignment, and variants between transcripts were identified (“called”) using FreeBayes. The total sequence number of wild-type (A) and *GNAQ*^Q209L^ (T) at position 626 was quantified using the “chromosome” view for each sample. The data shown are compiled from five independent experiments. This assay was validated using an independent digital-droplet-mutation-specific PCR assay manufactured by BioRad Laboratories (Hercules, CA, USA; Assay ID: dHsaMDV2010051).

### rAAV serotype assay

The self-complementary (sc) format of rAAV-CMV-GFP serotypes 1, 2, 3, 4, 5, 6, 8, and 9 were purchased from the UNC Vector Core (UNC-Chapel Hill, Chapel Hill, NC, USA) and are previously characterized^59^; 7.5×10^4^ cells for each UVM cell line were seeded in quadruplicate in 24-well plates, treated with 1.0×10^4^ vg/cell the following day, and monitored for GFP expression. Three days post-transduction, cells were treated with 0.05% trypsin-EDTA (Gibco/Life Technologies, Grand Island, NY, USA), resuspended in cell culture media, and fixed with 3.7% formaldehyde. GFP fluorescence was measured on the Thermo Fisher Attune NxT flow cytometer (Waltham, MA, USA) maintained by the UNC Flow Cytometry Core (UNC-Chapel Hill, Chapel Hill, NC, USA). Analysis was performed using the FlowJo Software (Version 10 for Windows 10, Ashland, Oregon, USA).

### rAAV production and characterization

rAAV-shGNAQ^Q209L^ and rAAV-shNTC were designed as described previously and displayed in Figure 5A. rAAV plasmid sequences were submitted to VectorBuilder Inc. (Chicago, IL, USA) for the manufacture of *in vitro*-grade preparations of rAAV. rAAV preparations were independently characterized via alkaline gel electrophoresis and SYBR Gold staining to confirm vector genome size and quantitative PCR (Figure S5) with a custom TaqMan primer/probe set targeting the CMV promoter to confirm viral genome titer (Figure S4; Table S1).

### rAAV-based alamarBlue assay

1.0×10^4^ cells were seeded in 24-well plates, and the following day, 1.0×10^4^ vg/cell of rAAV2-shGNAQ^Q209L^ or rAAV2-shNTC were added to wells. An equivalent volume of PBS was used as a vehicle control. Plates were monitored for cell growth, and 7 days later, alamarBlue Viability Reagent was added to a final concentration of 10%. Plates were analyzed in the manner described above for the siRNA-based alamarBlue assay. Data shown are a compilation of at least three independent experiments with at least four replicates each.

### rAAV-based clonogenic survival assay

1.0×10^3^ single cells were seeded in 10-cm plates, and the following day, 1.0×10^4^ vg/cell of either rAAV2-shGNAQ^Q209L^ or rAAV2-shNTC were added to plates. An equivalent volume of PBS was used as a vehicle control. Plates were fed with fresh media every 2–3 days. Fourteen days post-transduction, plates were stained and counted as previously described for siRNA-based clonogenic survival. Data shown are a compilation of at least two independent experiments with four to six replicates each.

### Figure generation and data analysis

Data were compiled and analyzed in Microsoft Excel for Windows 10 (Redmond, WA, USA) and/or GraphPad Prism version 10 for Windows (GraphPad Software, Boston, MA, USA), unless otherwise noted. Visualizations of data were created using GraphPad Prism 10 and Adobe Illustrator 2024 (Adobe, San Jose, CA, USA). Figure 5A was generated using Biorender.com. An unpaired t test was used to determine statistical significance between the means of experimental groups, and differences were considered significant if the *p* value <0.05. Averages are represented as the mean of the dataset, and statistical error is displayed as ± the standard deviation (SD) of the mean. Data were normalized to either NTC siRNA or PBS data for accurate comparisons among cell lines.

## Data availability

Raw data files are available upon request from the corresponding author.

## Acknowledgments

This work was directly supported by the North Carolina Collaboratory, the Murray Ocular Melanoma Fund, the UNC-Chapel Hill Office of the Provost’s IBM Junior Faculty Career Development Award (Jacquelyn J. Bower), and the NC TraCS Institute Pilot Award 550KR262105. The authors would like to thank the UNC Vector Core/R. Jude Samulski for scAAV preparations used in this study. The authors would also like to thank Dr. Martine Jeager of Leiden University for the Mel285 cell line. Flow cytometry data were generated with assistance from the UNC Flow Cytometry Core Facility (RRID:SCR_019170), supported in part by P30 CA016086 Cancer Center Core Support Grant to the UNC Lineberger Comprehensive Cancer Center, the North Carolina Biotech Center Institutional Support Grant 2012-IDG-1006, the North Carolina Biotech Center Institutional Support Grant 2017-IDG-1025, and by the 10.13039/100000002National Institutes of Health 1UM2AI30836-01. The content is solely the responsibility of the authors and does not necessarily represent the official views of the National Institutes of Health.

## Author contributions

M.L.H. and J.J.B. conceived and designed the study and secured its funding. T.F.M., E.J.S., J.D., and J.J.B. performed the experiments and collected data. T.F.M., E.J.S., J.D., M.L.H., and J.J.B. performed data analysis and interpretation. T.F.M., M.L.H., and J.J.B. wrote, reviewed, and edited the manuscript.

## Declaration of interests

T.F.M., M.L.H., and J.J.B. are co-inventors of presented technology and hold part ownership of submitted patent U.S. Provisional Application No. 63/650,097. The remaining authors declare no competing interests.

## Supplemental information

Document S1. Figures S1–S5 and Table S1

Document S2. Article plus supplemental information

## References

[1] Singh A.D., Turell M.E., Topham A.K. (2011). Uveal melanoma: trends in incidence, treatment, and survival. Ophthalmology.

[2] Chang A.E., Karnell L.H., Menck H.R. (1998). The National Cancer Data Base report on cutaneous and noncutaneous melanoma: a summary of 84,836 cases from the past decade. Cancer.

[3] McLaughlin C.C., Wu X.C., Jemal A., Martin H.J., Roche L.M., Chen V.W. (2005). Incidence of noncutaneous melanomas in the U.S. Cancer.

[4] Damato E.M., Damato B.E. (2012). Detection and time to treatment of uveal melanoma in the United Kingdom: an evaluation of 2,384 patients. Ophthalmology.

[5] Finger P.T. (2024). Yttrium-90 Episcleral Plaque Brachytherapy for Choroidal Melanoma. J. Vitreoretin. Dis..

[6] Collaborative Ocular Melanoma Study Group (2006). The COMS randomized trial of iodine 125 brachytherapy for choroidal melanoma: V. Twelve-year mortality rates and prognostic factors: COMS report No. 28. Arch. Ophthalmol..

[7] Bechrakis N.E., Bornfeld N., Zöller I., Foerster M.H. (2002). Iodine 125 plaque brachytherapy versus transscleral tumor resection in the treatment of large uveal melanomas. Ophthalmology.

[8] Kujala E., Mäkitie T., Kivelä T. (2003). Very long-term prognosis of patients with malignant uveal melanoma. Investig. Ophthalmol. Vis. Sci..

[9] Rietschel P., Panageas K.S., Hanlon C., Patel A., Abramson D.H., Chapman P.B. (2005). Variates of survival in metastatic uveal melanoma. J. Clin. Oncol..

[10] Augsburger J.J., Corrêa Z.M., Shaikh A.H. (2009). Effectiveness of treatments for metastatic uveal melanoma. Am. J. Ophthalmol..

[11] Kuk D., Shoushtari A.N., Barker C.A., Panageas K.S., Munhoz R.R., Momtaz P., Ariyan C.E., Brady M.S., Coit D.G., Bogatch K. (2016). Prognosis of Mucosal, Uveal, Acral, Nonacral Cutaneous, and Unknown Primary Melanoma From the Time of First Metastasis. Oncologist.

[12] Jensen O.A. (1982). Malignant melanomas of the human uvea: 25-year follow-up of cases in Denmark, 1943--1952. Acta Ophthalmol..

[13] ICGC/TCGA Pan-Cancer Analysis of Whole Genomes Consortium (2020). Pan-cancer analysis of whole genomes. Nature.

[14] Van Raamsdonk C.D., Bezrookove V., Green G., Bauer J., Gaugler L., O'Brien J.M., Simpson E.M., Barsh G.S., Bastian B.C. (2009). Frequent somatic mutations of GNAQ in uveal melanoma and blue naevi. Nature.

[15] Van Raamsdonk C.D., Griewank K.G., Crosby M.B., Garrido M.C., Vemula S., Wiesner T., Obenauf A.C., Wackernagel W., Green G., Bouvier N. (2010). Mutations in GNA11 in uveal melanoma. N. Engl. J. Med..

[16] Onken M.D., Makepeace C.M., Kaltenbronn K.M., Kanai S.M., Todd T.D., Wang S., Broekelmann T.J., Rao P.K., Cooper J.A., Blumer K.J. (2018). Targeting nucleotide exchange to inhibit constitutively active G protein alpha subunits in cancer cells. Sci. Signal..

[17] Neves S.R., Ram P.T., Iyengar R. (2002). G protein pathways. Science.

[18] Dorsam R.T., Gutkind J.S. (2007). G-protein-coupled receptors and cancer. Nat. Rev. Cancer.

[19] Oldham W.M., Hamm H.E. (2008). Heterotrimeric G protein activation by G-protein-coupled receptors. Nat. Rev. Mol. Cell Biol..

[20] Kalinec G., Nazarali A.J., Hermouet S., Xu N., Gutkind J.S. (1992). Mutated alpha subunit of the Gq protein induces malignant transformation in NIH 3T3 cells. Mol. Cell Biol..

[21] Landis C.A., Masters S.B., Spada A., Pace A.M., Bourne H.R., Vallar L. (1989). GTPase inhibiting mutations activate the alpha chain of Gs and stimulate adenylyl cyclase in human pituitary tumours. Nature.

[22] Feng X., Degese M.S., Iglesias-Bartolome R., Vaque J.P., Molinolo A.A., Rodrigues M., Zaidi M.R., Ksander B.R., Merlino G., Sodhi A. (2014). Hippo-independent activation of YAP by the GNAQ uveal melanoma oncogene through a trio-regulated rho GTPase signaling circuitry. Cancer Cell.

[23] Yu F.X., Luo J., Mo J.S., Liu G., Kim Y.C., Meng Z., Zhao L., Peyman G., Ouyang H., Jiang W. (2014). Mutant Gq/11 promote uveal melanoma tumorigenesis by activating YAP. Cancer Cell.

[24] Sudol M., Bork P., Einbond A., Kastury K., Druck T., Negrini M., Huebner K., Lehman D. (1995). Characterization of the mammalian YAP (Yes-associated protein) gene and its role in defining a novel protein module, the WW domain. J. Biol. Chem..

[25] Zhao B., Li L., Lei Q., Guan K.L. (2010). The Hippo-YAP pathway in organ size control and tumorigenesis: an updated version. Genes Dev..

[26] Dong J., Feldmann G., Huang J., Wu S., Zhang N., Comerford S.A., Gayyed M.F., Anders R.A., Maitra A., Pan D. (2007). Elucidation of a universal size-control mechanism in Drosophila and mammals. Cell.

[27] Li H., Li Q., Dang K., Ma S., Cotton J.L., Yang S., Zhu L.J., Deng A.C., Ip Y.T., Johnson R.L. (2019). YAP/TAZ Activation Drives Uveal Melanoma Initiation and Progression. Cell Rep..

[28] Yu F.X., Zhao B., Panupinthu N., Jewell J.L., Lian I., Wang L.H., Zhao J., Yuan H., Tumaneng K., Li H. (2012). Regulation of the Hippo-YAP pathway by G-protein-coupled receptor signaling. Cell.

[29] Zhao B., Ye X., Yu J., Li L., Li W., Li S., Yu J., Lin J.D., Wang C.Y., Chinnaiyan A.M. (2008). TEAD mediates YAP-dependent gene induction and growth control. Genes Dev..

[30] Fujioka M., Koda S., Morimoto Y., Biemann K. (1988). Structure of FR900359, a cyclic depsipeptide from Ardisia crenata sims. J. Org. Chem..

[31] Lapadula D., Farias E., Randolph C.E., Purwin T.J., McGrath D., Charpentier T.H., Zhang L., Wu S., Terai M., Sato T. (2019). Effects of Oncogenic Galpha(q) and Galpha(11) Inhibition by FR900359 in Uveal Melanoma. Mol. Cancer Res..

[32] Kawasaki T., Taniguchi M., Moritani Y., Uemura T., Shigenaga T., Takamatsu H., Hayashi K., Takasaki J., Saito T., Nagai K. (2005). Pharmacological properties of YM-254890, a specific G(alpha)q/11 inhibitor, on thrombosis and neointima formation in mice. Thromb. Haemost..

[33] Takasaki J., Saito T., Taniguchi M., Kawasaki T., Moritani Y., Hayashi K., Kobori M. (2004). A novel Galphaq/11-selective inhibitor. J. Biol. Chem..

[34] Hitchman T.D., Bayshtok G., Ceraudo E., Moore A.R., Lee C., Jia R., Wang N., Pachai M.R., Shoushtari A.N., Francis J.H. (2021). Combined Inhibition of Galpha(q) and MEK Enhances Therapeutic Efficacy in Uveal Melanoma. Clin. Cancer Res..

[35] Offermanns S., Zhao L.P., Gohla A., Sarosi I., Simon M.I., Wilkie T.M. (1998). Embryonic cardiomyocyte hypoplasia and craniofacial defects in G alpha q/G alpha 11-mutant mice. EMBO J..

[36] Onken M.D., Makepeace C.M., Kaltenbronn K.M., Choi J., Hernandez-Aya L., Weilbaecher K.N., Piggott K.D., Rao P.K., Yuede C.M., Dixon A.J. (2021). Targeting primary and metastatic uveal melanoma with a G protein inhibitor. J. Biol. Chem..

[37] Schlegel J.G., Tahoun M., Seidinger A., Voss J.H., Kuschak M., Kehraus S., Schneider M., Matthey M., Fleischmann B.K., König G.M. (2021). Macrocyclic Gq Protein Inhibitors FR900359 and/or YM-254890-Fit for Translation?. ACS Pharmacol. Transl. Sci..

[38] Hassel J.C., Berking C., Forschner A., Gebhardt C., Heinzerling L., Meier F., Ochsenreither S., Siveke J., Hauschild A., Schadendorf D. (2023). Practical guidelines for the management of adverse events of the T cell engager bispecific tebentafusp. Eur. J. Cancer.

[39] Hassel J.C., Piperno-Neumann S., Rutkowski P., Baurain J.F., Schlaak M., Butler M.O., Sullivan R.J., Dummer R., Kirkwood J.M., Orloff M. (2023). Three-Year Overall Survival with Tebentafusp in Metastatic Uveal Melanoma. N. Engl. J. Med..

[40] Nathan P., Hassel J.C., Rutkowski P., Baurain J.F., Butler M.O., Schlaak M., Sullivan R.J., Ochsenreither S., Dummer R., Kirkwood J.M. (2021). Overall Survival Benefit with Tebentafusp in Metastatic Uveal Melanoma. N. Engl. J. Med..

[41] Gelmi M.C., Jager M.J. (2024). Uveal melanoma: Current evidence on prognosis, treatment and potential developments. Asia. Pac. J. Ophthalmol..

[42] Schwarz D.S., Ding H., Kennington L., Moore J.T., Schelter J., Burchard J., Linsley P.S., Aronin N., Xu Z., Zamore P.D. (2006). Designing siRNA that distinguish between genes that differ by a single nucleotide. PLoS Genet..

[43] Elbashir S.M., Harborth J., Lendeckel W., Yalcin A., Weber K., Tuschl T. (2001). Duplexes of 21-nucleotide RNAs mediate RNA interference in cultured mammalian cells. Nature.

[44] Paddison P.J., Caudy A.A., Hannon G.J. (2002). Stable suppression of gene expression by RNAi in mammalian cells. Proc. Natl. Acad. Sci. USA.

[45] Izquierdo M. (2005). Short interfering RNAs as a tool for cancer gene therapy. Cancer Gene Ther..

[46] Ambrosini G., Musi E., Ho A.L., de Stanchina E., Schwartz G.K. (2013). Inhibition of mutant GNAQ signaling in uveal melanoma induces AMPK-dependent autophagic cell death. Mol. Cancer Ther..

[47] Ksander B.R., Rubsamen P.E., Olsen K.R., Cousins S.W., Streilein J.W. (1991). Studies of tumor-infiltrating lymphocytes from a human choroidal melanoma. Investig. Ophthalmol. Vis. Sci..

[48] Griewank K.G., Yu X., Khalili J., Sozen M.M., Stempke-Hale K., Bernatchez C., Wardell S., Bastian B.C., Woodman S.E. (2012). Genetic and molecular characterization of uveal melanoma cell lines. Pigment Cell Melanoma Res..

[49] Krampe B., Al-Rubeai M. (2010). Cell death in mammalian cell culture: molecular mechanisms and cell line engineering strategies. Cytotechnology.

[50] Carton R.J., Doyle M.G., Kearney H., Steward C.A., Lench N.J., Rogers A., Heinzen E.L., McDonald S., Fay J., Lacey A. (2024). Somatic variants as a cause of drug-resistant epilepsy including mesial temporal lobe epilepsy with hippocampal sclerosis. Epilepsia.

[51] Verbik D.J., Murray T.G., Tran J.M., Ksander B.R. (1997). Melanomas that develop within the eye inhibit lymphocyte proliferation. Int. J. Cancer.

[52] De Waard-Siebinga I., Blom D.J., Griffioen M., Schrier P.I., Hoogendoorn E., Beverstock G., Danen E.H., Jager M.J. (1995). Establishment and characterization of an uveal-melanoma cell line. Int. J. Cancer.

[53] Hickerson R.P., Vlassov A.V., Wang Q., Leake D., Ilves H., Gonzalez-Gonzalez E., Contag C.H., Johnston B.H., Kaspar R.L. (2008). Stability study of unmodified siRNA and relevance to clinical use. Oligonucleotides.

[54] Czauderna F., Fechtner M., Dames S., Aygün H., Klippel A., Pronk G.J., Giese K., Kaufmann J. (2003). Structural variations and stabilising modifications of synthetic siRNAs in mammalian cells. Nucleic Acids Res..

[55] Holen T., Amarzguioui M., Wiiger M.T., Babaie E., Prydz H. (2002). Positional effects of short interfering RNAs targeting the human coagulation trigger Tissue Factor. Nucleic Acids Res..

[56] Sato N., Saga Y., Uchibori R., Tsukahara T., Urabe M., Kume A., Fujiwara H., Suzuki M., Ozawa K., Mizukami H. (2018). Eradication of cervical cancer in vivo by an AAV vector that encodes shRNA targeting human papillomavirus type 16 E6/E7. Int. J. Oncol..

[57] Bower J.J., Song L., Bastola P., Hirsch M.L. (2021). Harnessing the Natural Biology of Adeno-Associated Virus to Enhance the Efficacy of Cancer Gene Therapy. Viruses.

[58] Issa S.S., Shaimardanova A.A., Solovyeva V.V., Rizvanov A.A. (2023). Various AAV Serotypes and Their Applications in Gene Therapy: An Overview. Cells.

[59] McCarty D.M., Monahan P.E., Samulski R.J. (2001). Self-complementary recombinant adeno-associated virus (scAAV) vectors promote efficient transduction independently of DNA synthesis. Gene Ther..

[60] Ma H., Wu Y., Dang Y., Choi J.G., Zhang J., Wu H. (2014). Pol III Promoters to Express Small RNAs: Delineation of Transcription Initiation. Mol. Ther. Nucleic Acids.

[61] Sayers E.W., Bolton E.E., Brister J.R., Canese K., Chan J., Comeau D.C., Connor R., Funk K., Kelly C., Kim S. (2022). Database resources of the national center for biotechnology information. Nucleic Acids Res..

[62] Ando H., Niki Y., Yoshida M., Ito M., Akiyama K., Kim J.H., Yoon T.J., Matsui M.S., Yarosh D.B., Ichihashi M. (2011). Involvement of pigment globules containing multiple melanosomes in the transfer of melanosomes from melanocytes to keratinocytes. Cell. Logist..

[63] Lu F., Yan D., Zhou X., Hu D.N., Qu J. (2007). Expression of melanin-related genes in cultured adult human retinal pigment epithelium and uveal melanoma cells. Mol. Vis..

[64] Diener-West M., Reynolds S.M., Agugliaro D.J., Caldwell R., Cumming K., Earle J.D., Green D.L., Hawkins B.S., Hayman J., Jaiyesimi I. (2004). Screening for metastasis from choroidal melanoma: the Collaborative Ocular Melanoma Study Group Report 23. J. Clin. Oncol..

[65] Collaborative Ocular Melanoma Study Group (2001). Assessment of metastatic disease status at death in 435 patients with large choroidal melanoma in the Collaborative Ocular Melanoma Study (COMS): COMS report no. 15. Arch. Ophthalmol..

[66] Carvajal R.D., Schwartz G.K., Tezel T., Marr B., Francis J.H., Nathan P.D. (2017). Metastatic disease from uveal melanoma: treatment options and future prospects. Br. J. Ophthalmol..

[67] Croce M., Ferrini S., Pfeffer U., Gangemi R. (2019). Targeted Therapy of Uveal Melanoma: Recent Failures and New Perspectives. Cancers (Basel).

[68] Bongianino R., Denegri M., Mazzanti A., Lodola F., Vollero A., Boncompagni S., Fasciano S., Rizzo G., Mangione D., Barbaro S. (2017). Allele-Specific Silencing of Mutant mRNA Rescues Ultrastructural and Arrhythmic Phenotype in Mice Carriers of the R4496C Mutation in the Ryanodine Receptor Gene (RYR2). Circ. Res..

[69] Miller V.M., Xia H., Marrs G.L., Gouvion C.M., Lee G., Davidson B.L., Paulson H.L. (2003). Allele-specific silencing of dominant disease genes. Proc. Natl. Acad. Sci. USA.

[70] Noguchi S., Ogawa M., Kawahara G., Malicdan M.C., Nishino I. (2014). Allele-specific Gene Silencing of Mutant mRNA Restores Cellular Function in Ullrich Congenital Muscular Dystrophy Fibroblasts. Mol. Ther. Nucleic Acids.

[71] Romano R., De Luca M., Del Fiore V.S., Pecoraro M., Lattante S., Sabatelli M., La Bella V., Bucci C. (2022). Allele-specific silencing as therapy for familial amyotrophic lateral sclerosis caused by the p.G376D TARDBP mutation. Brain Commun..

[72] Hickerson R.P., Smith F.J.D., Reeves R.E., Contag C.H., Leake D., Leachman S.A., Milstone L.M., McLean W.H.I., Kaspar R.L. (2008). Single-nucleotide-specific siRNA targeting in a dominant-negative skin model. J. Invest. Dermatol..

[73] Kobayashi Y., Fukuhara D., Akase D., Aida M., Ui-Tei K. (2022). siRNA Seed Region Is Divided into Two Functionally Different Domains in RNA Interference in Response to 2'-OMe Modifications. ACS Omega.

[74] Kobayashi Y., Tian S., Ui-Tei K. (2022). The siRNA Off-Target Effect Is Determined by Base-Pairing Stabilities of Two Different Regions with Opposite Effects. Genes.

[75] Anderson E.M., Birmingham A., Baskerville S., Reynolds A., Maksimova E., Leake D., Fedorov Y., Karpilow J., Khvorova A. (2008). Experimental validation of the importance of seed complement frequency to siRNA specificity. RNA.

[76] Jackson A.L., Burchard J., Schelter J., Chau B.N., Cleary M., Lim L., Linsley P.S. (2006). Widespread siRNA "off-target" transcript silencing mediated by seed region sequence complementarity. RNA.

[77] Dana H., Chalbatani G.M., Mahmoodzadeh H., Karimloo R., Rezaiean O., Moradzadeh A., Mehmandoost N., Moazzen F., Mazraeh A., Marmari V. (2017). Molecular Mechanisms and Biological Functions of siRNA. Int. J. Biomed. Sci..

[78] Offermanns S., Hashimoto K., Watanabe M., Sun W., Kurihara H., Thompson R.F., Inoue Y., Kano M., Simon M.I. (1997). Impaired motor coordination and persistent multiple climbing fiber innervation of cerebellar Purkinje cells in mice lacking Galphaq. Proc. Natl. Acad. Sci. USA.

[79] Offermanns S., Toombs C.F., Hu Y.H., Simon M.I. (1997). Defective platelet activation in G alpha(q)-deficient mice. Nature.

[80] Khan A.A., Betel D., Miller M.L., Sander C., Leslie C.S., Marks D.S. (2009). Transfection of small RNAs globally perturbs gene regulation by endogenous microRNAs. Nat. Biotechnol..

[81] Onken M.D., Noda S.E., Kaltenbronn K.M., Frankfater C., Makepeace C.M., Fettig N., Piggott K.D., Custer P.L., Ippolito J.E., Blumer K.J. (2022). Oncogenic Gq/11 signaling acutely drives and chronically sustains metabolic reprogramming in uveal melanoma. J. Biol. Chem..

[82] Jager M.J., Magner J.A.B., Ksander B.R., Dubovy S.R. (2016). Uveal Melanoma Cell Lines: Where do they come from? (An American Ophthalmological Society Thesis). Trans. Am. Ophthalmol. Soc..

[83] Palliser D., Chowdhury D., Wang Q.Y., Lee S.J., Bronson R.T., Knipe D.M., Lieberman J. (2006). An siRNA-based microbicide protects mice from lethal herpes simplex virus 2 infection. Nature.

[84] Zimmermann T.S., Lee A.C.H., Akinc A., Bramlage B., Bumcrot D., Fedoruk M.N., Harborth J., Heyes J.A., Jeffs L.B., John M. (2006). RNAi-mediated gene silencing in non-human primates. Nature.

[85] Mantei A., Rutz S., Janke M., Kirchhoff D., Jung U., Patzel V., Vogel U., Rudel T., Andreou I., Weber M., Scheffold A. (2008). siRNA stabilization prolongs gene knockdown in primary T lymphocytes. Eur. J. Immunol..

[86] Bartlett D.W., Davis M.E. (2006). Insights into the kinetics of siRNA-mediated gene silencing from live-cell and live-animal bioluminescent imaging. Nucleic Acids Res..

[87] Traber G.M., Yu A.-M. (2024). The Growing Class of Novel RNAi Therapeutics. Mol. Pharmacol..

[88] Administration U.S.F.A.D. (2024). Approved Cellular and Gene Therapy Products. https://www.fda.gov/vaccines-blood-biologics/cellular-gene-therapy-products/approved-cellular-and-gene-therapy-products.

[89] Chancellor D., Barrett D., Nguyen-Jatkoe L., Millington S., Eckhardt F. (2023). The state of cell and gene therapy in 2023. Mol. Ther..

[90] Li C., Samulski R.J. (2020). Engineering adeno-associated virus vectors for gene therapy. Nat. Rev. Genet..

[91] Wu Z., Asokan A., Samulski R.J. (2006). Adeno-associated virus serotypes: vector toolkit for human gene therapy. Mol. Ther..

[92] Brummelkamp T.R., Bernards R., Agami R. (2002). A system for stable expression of short interfering RNAs in mammalian cells. Science.

[93] Tomar R.S., Matta H., Chaudhary P.M. (2003). Use of adeno-associated viral vector for delivery of small interfering RNA. Oncogene.

[94] Nguyen G.N., Everett J.K., Kafle S., Roche A.M., Raymond H.E., Leiby J., Wood C., Assenmacher C.A., Merricks E.P., Long C.T. (2021). A long-term study of AAV gene therapy in dogs with hemophilia A identifies clonal expansions of transduced liver cells. Nat. Biotechnol..

[95] Nathwani A.C., Rosales C., McIntosh J., Rastegarlari G., Nathwani D., Raj D., Nawathe S., Waddington S.N., Bronson R., Jackson S. (2011). Long-term safety and efficacy following systemic administration of a self-complementary AAV vector encoding human FIX pseudotyped with serotype 5 and 8 capsid proteins. Mol. Ther..

[96] Dorsett Y., Tuschl T. (2004). siRNAs: applications in functional genomics and potential as therapeutics. Nat. Rev. Drug Discov..

[97] Hoek K., Rimm D.L., Williams K.R., Zhao H., Ariyan S., Lin A., Kluger H.M., Berger A.J., Cheng E., Trombetta E.S. (2004). Expression profiling reveals novel pathways in the transformation of melanocytes to melanomas. Cancer Res..

[98] Summerford C., Samulski R.J. (1998). Membrane-associated heparan sulfate proteoglycan is a receptor for adeno-associated virus type 2 virions. J. Virol..

[99] Qing K., Mah C., Hansen J., Zhou S., Dwarki V., Srivastava A. (1999). Human fibroblast growth factor receptor 1 is a co-receptor for infection by adeno-associated virus 2. Nat. Med..

[100] Sheppard H.M., Ussher J.E., Verdon D., Chen J., Taylor J.A., Dunbar P.R. (2013). Recombinant adeno-associated virus serotype 6 efficiently transduces primary human melanocytes. PLoS One.

[101] Pinto C., Silva G., Ribeiro A.S., Oliveira M., Garrido M., Bandeira V.S., Nascimento A., Coroadinha A.S., Peixoto C., Barbas A. (2019). Evaluation of AAV-mediated delivery of shRNA to target basal-like breast cancer genetic vulnerabilities. J. Biotechnol..

[102] Raj K., Ogston P., Beard P. (2001). Virus-mediated killing of cells that lack p53 activity. Nature.

[103] Fragkos M., Beard P. (2011). Mitotic catastrophe occurs in the absence of apoptosis in p53-null cells with a defective G1 checkpoint. PLoS One.

[104] Alam S., Bowser B.S., Conway M.J., Israr M., Tandon A., Meyers C. (2011). Adeno-associated virus type 2 infection activates caspase dependent and independent apoptosis in multiple breast cancer lines but not in normal mammary epithelial cells. Mol. Cancer.

[105] de la Maza L.M., Carter B.J. (1981). Inhibition of adenovirus oncogenicity in hamsters by adeno-associated virus DNA. J. Natl. Cancer Inst..

[106] Hirsch M.L., Fagan B.M., Dumitru R., Bower J.J., Yadav S., Porteus M.H., Pevny L.H., Samulski R.J. (2011). Viral single-strand DNA induces p53-dependent apoptosis in human embryonic stem cells. PLoS One.

[107] Bockstael O., Melas C., Pythoud C., Levivier M., McCarty D., Samulski R.J., De Witte O., Tenenbaum L. (2012). Rapid transgene expression in multiple precursor cell types of adult rat subventricular zone mediated by adeno-associated type 1 vectors. Hum. Gene Ther..

[108] Hordeaux J., Buza E.L., Dyer C., Goode T., Mitchell T.W., Richman L., Denton N., Hinderer C., Katz N., Schmid R. (2020). Adeno-Associated Virus-Induced Dorsal Root Ganglion Pathology. Hum. Gene Ther..

[109] Johnston S., Parylak S.L., Kim S., Mac N., Lim C., Gallina I., Bloyd C., Newberry A., Saavedra C.D., Novak O. (2021). AAV ablates neurogenesis in the adult murine hippocampus. eLife.

[110] Song L., Hasegawa T., Brown N.J., Bower J.J., Samulski R.J., Hirsch M.L. (2025). AAV vector transduction restriction and attenuated toxicity in hESCs via a rationally designed inverted terminal repeat. Nucleic Acids Res..

[111] Huang J.L.Y., Urtatiz O., Van Raamsdonk C.D. (2015). Oncogenic G Protein GNAQ Induces Uveal Melanoma and Intravasation in Mice. Cancer Res..

[112] Livak K.J., Schmittgen T.D. (2001). Analysis of relative gene expression data using real-time quantitative PCR and the 2(-Delta Delta C(T)) Method. Methods.

