# Supplementary material for: Allele-specific depletion of GNAQQ209L via siRNA or an rAAV2-shRNA vector induces selective toxicity in GNAQQ209L uveal melanoma cells
Source: Mol Ther Oncol. 2025 Jul 17;33(3):201020. doi: 10.1016/j.omton.2025.201020 (PMC12341522; doi:10.1016/j.omton.2025.201020)
Supplement: Document S1. Figures S1–S5 and Table S1 [file mmc1.pdf]

**OMTON, Volume *33***

## Supplemental information

# Allele-specific depletion of *GNAQ*$^{Q209L}$ via siRNA or an rAAV2-shRNA vector induces selective toxicity in *GNAQ*$^{Q209L}$ uveal melanoma cells

**Trace F. McCall, Emma J. Sawyer, Joshua Darnell, Matthew L. Hirsch, and Jacquelyn J. Bower**

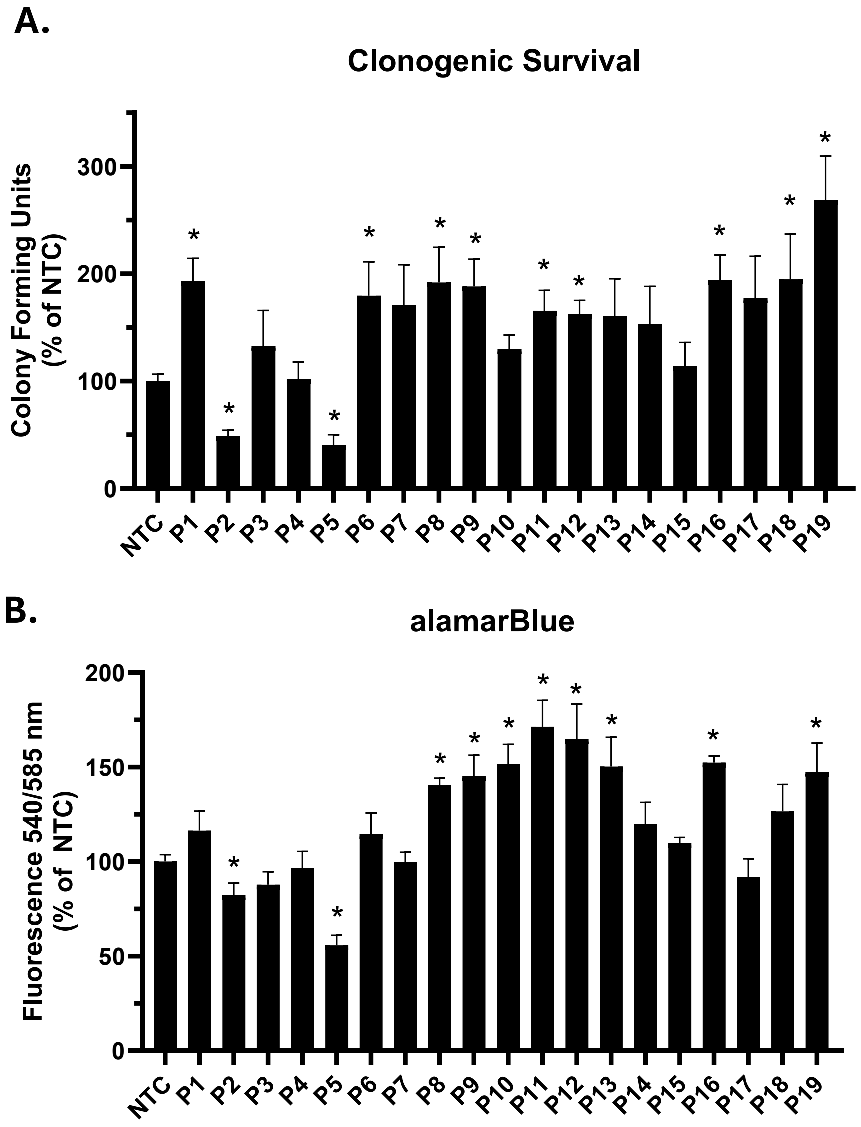


**Figure S1. P2 and P5 *GNAQ*$^{Q209L}$-targeting siRNAs reduce clonogenic survival and alamarBlue™ metabolic activity in a *GNAQ*$^{Q209L}$ UVM cell line**. The results of a non-targeting control siRNA (NTC) and the P1-P19 *GNAQ*$^{Q209L}$-targeting siRNA transfections on clonogenic survival (A) and alamarBlue™ metabolic activity (B) are displayed above. Solid bars represent the means of each data set. Error bars represent ± standard deviation of the mean. Transfection with only the P2 and the P5 sequences resulted in decreased viability of the Mel202 cell lines. Statistical significance was determined using an unpaired t-test. Significance levels are indicated by the following: no asterisk – not significant, $p > .05$; * - $p < .05$. All data presented are normalized to the mean of NTC siRNA.

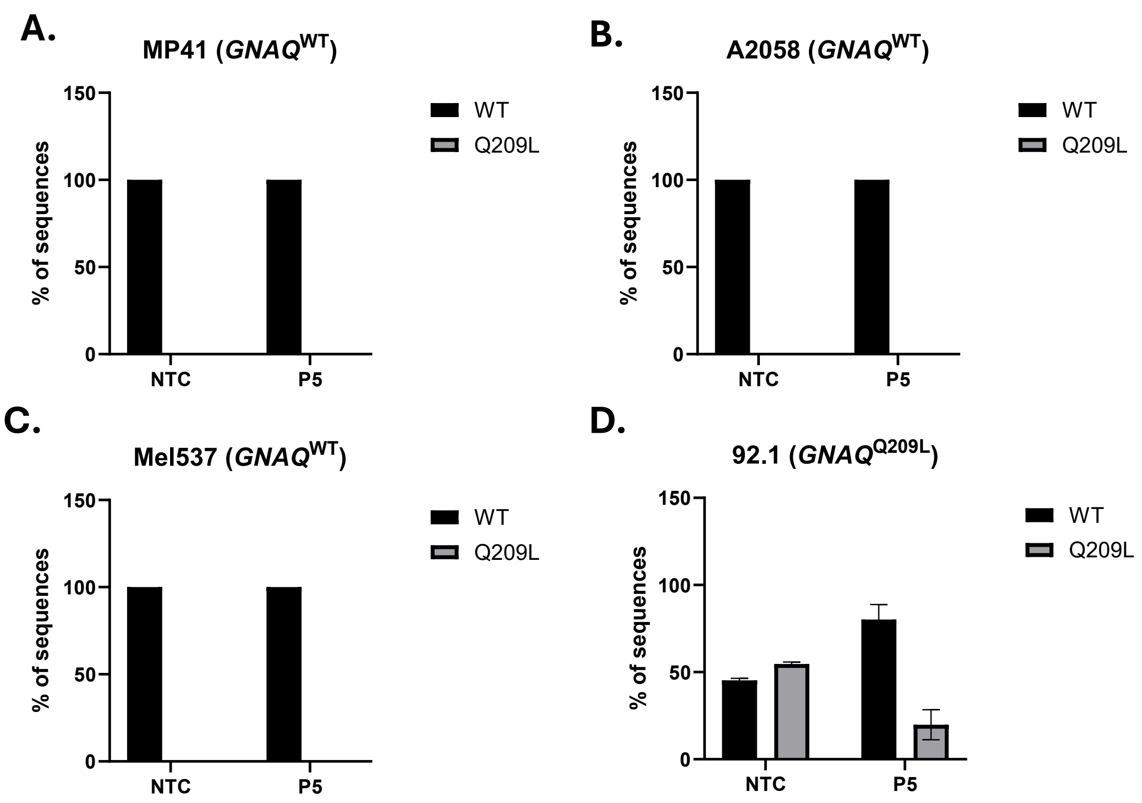


E.

| Cell Line (Mutation) | % of NGS Reads with *GNAQ*$^{\mathrm{WT}}$ | % of NGS Reads with GNAQ$^{\mathrm{Q209L}}$ | Ratio of *GNAQ*$^{\mathrm{WT}}$:*GNAQ*$^{\mathrm{Q209L}}$ Transcripts |
|---|---|---|---|
| MP41 (*GNAQ*$^{\mathrm{WT}}$) NTC siRNA | 99 | 0 | N/A |
| MP41 (*GNAQ*$^{\mathrm{WT}}$) P5 siRNA | 99 | 0 | N/A |
| A2058 (*GNAQ*$^{\mathrm{WT}}$) NTC siRNA | 99 | 0 | N/A |
| A2058 (*GNAQ*$^{\mathrm{WT}}$) P5 siRNA | 99 | 0 | N/A |
| Mel537 (*GNAQ*$^{\mathrm{WT}}$) NTC siRNA | 99 | 0 | N/A |
| Mel537 (*GNAQ*$^{\mathrm{WT}}$) P5 siRNA | 99 | 0 | N/A |
| 92.1 (*GNAQ*$^{\mathrm{Q209L}}$) NTC siRNA | 50 | 49 | 1:1 |
| 92.1 (*GNAQ*$^{\mathrm{Q209L}}$) P5 siRNA | 69 | 30 | 2.3:1 |

**Figure S2. Next-generation sequencing (NGS) to confirm allele-specific depletion via *GNAQ*$^{\mathrm{Q209L}}$-specific P5 siRNA is supported by a commercially available and wet-lab validated digital droplet PCR assay.** (A-D) A commercially available and wet-lab validated ddPCR GNAQ$^{\mathrm{Q209L}}$ (c.626 A>T) specific-assay (Bio-Rad Laboratories, Hercules, CA, USA; Assay ID: dHsaMDV2010051) was used to confirm the percentage of *GNAQ*$^{\mathrm{WT}}$ and *GNAQ*$^{\mathrm{Q209L}}$ sequences in NTC- and P5-transfected cell *GNAQ*$^{\mathrm{WT}}$ and *GNAQ*$^{\mathrm{Q209L}}$ cell lines, 24-hours post-transfection. (E) These data support the allele-specific quantification as completed via Amplicon-EZ NGS (Genewiz from Azenta, South Plainfield, NJ, USA) completed on these same samples.

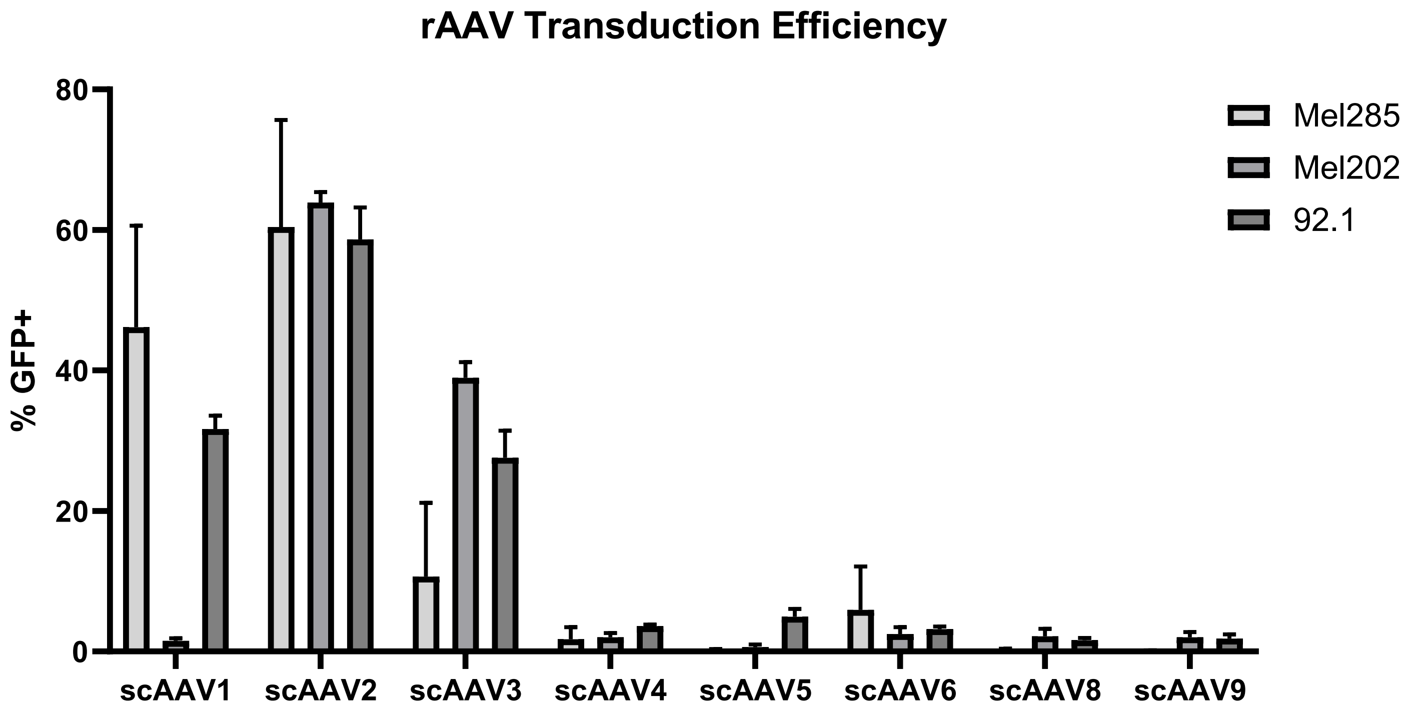


**Figure S3. AAV2 transduce three uveal melanoma cell lines with the highest efficiency.** The transduction efficiency of self-complementary (sc) adeno-associated virus (AAV) serotypes 1, 2, 3, 4, 5, 6, 8, and 9 in Mel285, Mel202, and 92.1 cells as measured through the % of GFP+ cells are displayed above. Cells were harvested and fixed for analysis three days following scAAV transduction. Data is the same as displayed in Figure 4A, with bars representing the means of each data set and error bars representing ± standard deviation of each mean.

A.

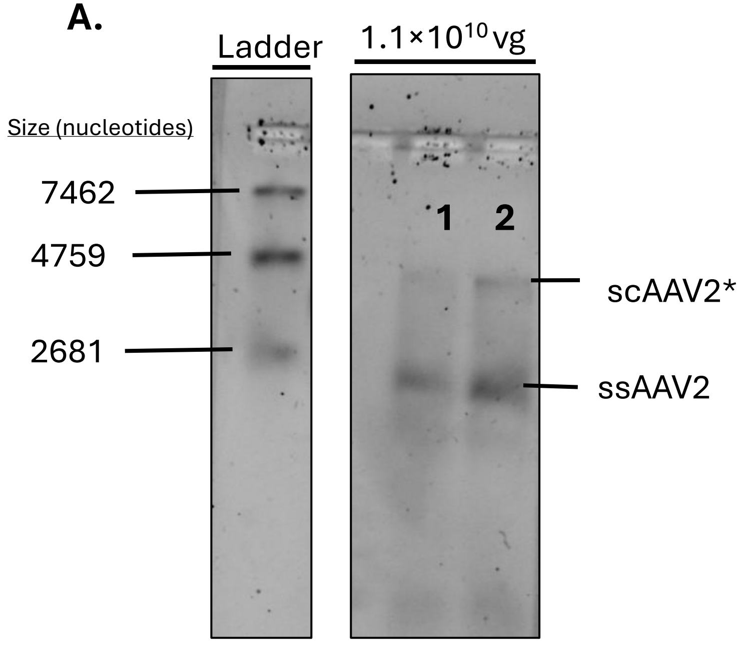


**(1)** ssAAV2-shNTC, 2225 nucleotides

**(2)** ssAAV2-shGNAQ$^{Q209L}$, 2210 nucleotides

B.

| **ssAAV2-shNTC** | **ssAAV2-shGNAQ$^{Q209L}$** |
|---|---|
| 5.58E+12 vg/mL | 5.98E+12 vg/mL |

**Figure S4. Characterization of single-stranded (ss) AAV preparations used *in vitro*.** (A) Roughly 1.1×10$^{10}$ vector genomes were loaded and run on an alkaline gel and stained with Sybr Gold to visualize the approximate size, in nucleotides, of each packaged genome. A ladder was also run consisting of DNA strands of size 7452, 4759, and 2681 nucleotides. Among both AAV2 vector preparations, the dominant species appears to be single-stranded. *However, another species appears at roughly double the size of the single-stranded packaged genome, indicating that some vector genomes were packaged as a self-complementary (sc) genome. (B) The viral genome (vg) titer of each preparation are displayed, quantified via qPCR with a customer primer/probe targeting the CMV promoter. Data shown are the means of three technical replicates each. (sh = short hairpin; NTC = non-targeting control)

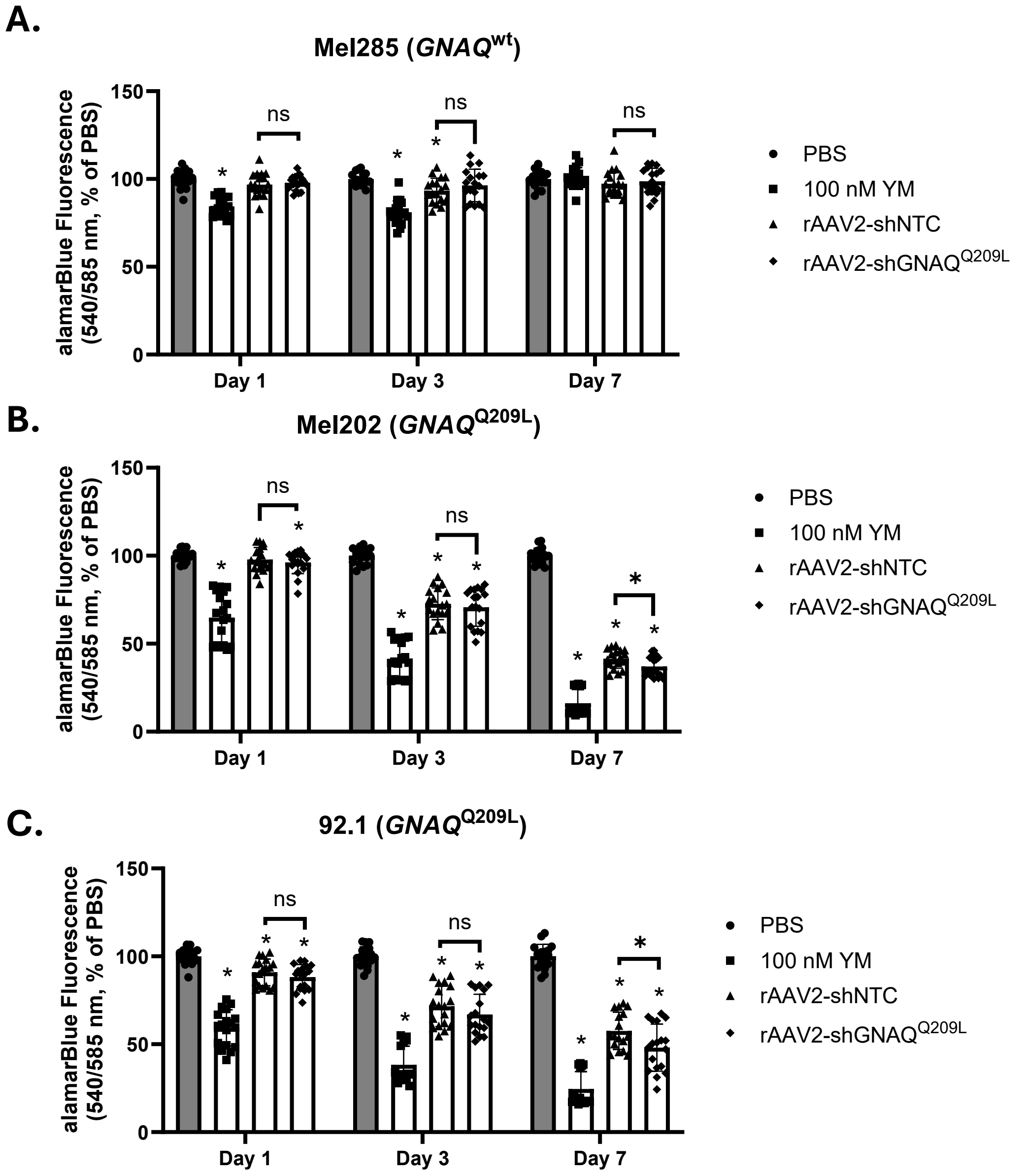


**Figure S5. rAAV2-shGNAQ$^{Q209L}$ –reduce alamarBlue fluorescence in *GNAQ*$^{Q209L}$ UVM cells in a time-specific manner compared to rAAV2-shNTC.** Mel285, Mel202, and 92.1 UVM cells were seeded at $2.0{\times}10^3$ cells/well in a 96-well plate. The following day, $1.0{\times}10^4$ vg/cell of either rAAV2-shNTC or rAAV2-shGNAQ$^{Q209L}$, 100 nM YM-254890 (YM; a positive control small molecule inhibitor of $G\alpha_{q/11}$), or an equivalent volume of phosphate-buffered saline (PBS; vehicle control) were added to wells in replicates of six. This was repeated across three plates for each cell line, with each plate representing 1 day, 3 days, or 7 days post-transduction in which alamarBlue dye was added and fluorescence was measured, representing a quantification of metabolic activity of each well. Three individual experimental repeats were completed for each of the days measured and for each cell line. Statistical significance was determined using an unpaired t-test. Significance levels are indicated by the following: ns – not significant, $p > .05$; * - $p < .05$. All data presented are normalized to the mean of PBS-treated wells.

| Table S1. TaqMan primer/probe sets. | |
|---|---|
| **Target** | **TaqMan Assay Number or Custom Sequence** |
| *GNAQ* | Hs00387073_m1 |
| *CTGF* | Hs00170014_m1 |
| *CYR61* | Hs00155479_m1 |
| *GAPDH* | Hs02786624_g1 |
| CMV promoter | Forward: 5' –CCAAGTCTCCACCCCATTGAC – 3'<br>Reverse: 5' – GGCGGAGTTGTTACGACATTTTG – 3'<br>Probe: 5' – TTGGCAC – 3' |
